# Multiscale Conceptual Design of a Scalable and Sustainable Process to Dissolve and Regenerate Keratin from Chicken Feathers

**DOI:** 10.1021/acs.iecr.3c01843

**Published:** 2023-08-16

**Authors:** Víctor R. Ferro, Héctor Leiva, Erasmo Cadena, José Luis Valverde

**Affiliations:** † Department of Chemical Engineering, Universidad Autónoma de Madrid, 28049 Madrid, Spain; ‡ Department of Green Chemistry and Technology, Ghent University, 9000 Gent, Belgium; § Department of Chemical Engineering, Universidad de Castilla la Mancha, 13071 Ciudad Real, Spain

## Abstract

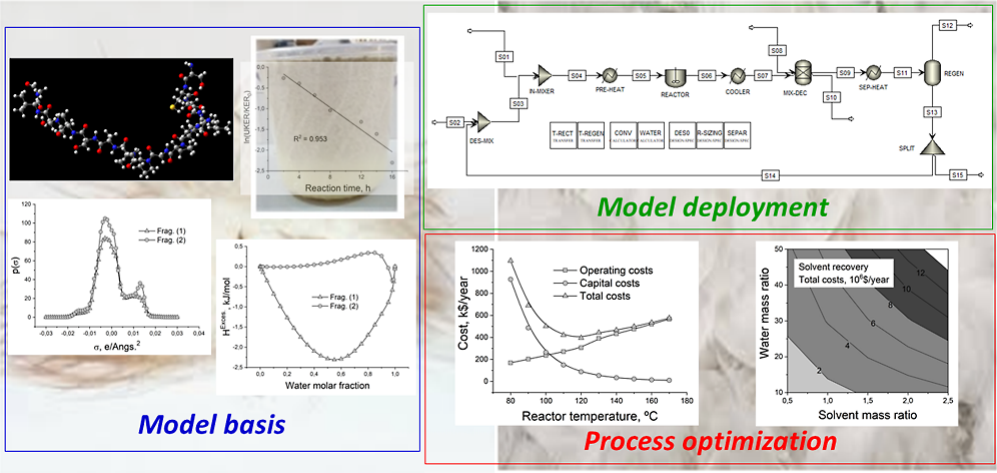

A multiscale strategy was used to conceptually design and economically analyze a scalable and sustainable process for dissolving and regenerating keratin from chicken feathers by using a sodium acetate–urea deep eutectic solvent as the reacting media. In this study, the recovery and recycling of the solvent were also considered. Moreover, molecular modeling of the solvent, keratin and its derivatives, property estimation of the corresponding mixtures, and simulation of the different process alternatives proposed, including the equipment sizing, estimation of energy needs, and economic analysis were presented. A quasi-planar cluster governed by H-bond interactions resulted in the most stable configuration of the deep eutectic solvent. Molecular models having molecular weights higher than 1.400 g/mol were created to represent the keratin species, where the most abundant amino acids in the feathers were included and conveniently ordered in the chain. Property estimations performed with the conductor-like screening model-real solvent succeeded in describing the main features of the interactions between the keratin derivatives and the solvents used. The process analysis performed on several alternatives showed that the process is technically and economically viable at the industrial scale, the costs being strongly dependent on the excess of both the solvent used to dissolve keratin and the water added for its regeneration. Several options to improve the process and reduce the costs are discussed.

## 1 Introduction

Chicken feather is a sort of biological waste, generated in large quantities by the poultry meat industry,^1,2^ which can cause environmental damage and disease transmission. The current treatment of the feathers consists mostly of incineration or landfilling.^1,2^ Small amounts are valorized as fertilizers or feather meal.^3,4^ Moreover, important efforts are being made to treat and transform it in products of higher added value.^3,5^ Poultry feathers contain more than 90% keratin, which can be used directly as such or transformed to valuable products of lower molecular weight. However, its peculiar structure^3,6–9^ makes keratin insoluble in water and other conventional solvents besides being inert to many chemical and biological treatments. This severely limits its processability and transformability into new products. Thus, the keratin supramolecular structure should be first broken or modified to enable further processing by enzymatic, biological, or other physicochemical methods.^5^

The supramolecular organization is one of the most relevant structural features of keratin.^3,10,11^ It is supported by a complete set of physicochemical interactions that include disulfide, ionic, and hydrogen bonds along with hydrophobic interactions of van der Waals and a dispersive nature.^3,9–12^ Disulfide bonds, which cross-link adjacent polypeptide chains, play a significant role in keratin’s molecular structure. Thus, breaking disulfide bonds is the key issue of the processes aimed at transforming keratin contained in chicken feathers into products of higher benefit.

Several chemical treatments have been evaluated as an alternative to solubilize and separate keratin and keratin derivatives from chicken feathers.^5^ In these methods, several reagents have been used to cleave the disulfide bonds by chemical reaction.^5,12–16^ However, most of these chemicals are toxic, difficult to recycle, and expensive to produce. In addition, some of them can attack the peptide bonds, causing protein degradation.^1,5,17^

Ionic liquids (ILs) have also been considered in the search of more specific solvents to dissolve the keratin contained in different raw materials.^18–26^ They are excellent candidates because their properties can be tuned by selecting or modifying properly the constituent cations and anions.^27^ Remarkably, ILs can selectively break disulfide bonds, preserving the protein backbone. Nevertheless, ILs are, as a rule, quite expensive, limiting the extension of this alternative to a commercial scale.^28^

Moreover, it has been shown that mixtures of certain solids having a specific composition, commonly named deep eutectic solvents (DESs), exhibit excellent solvent properties.^29,30^ Even mixtures having molar ratios of the components somewhat different to that of the eutectic can also show similar behavior.^31^ Due to these properties, they have found potential applications in different fields.^30,32–39^ More recently, the sodium acetate (NaAc)—urea and choline chloride (CholineCl)—urea DESs have been assessed as reacting media to dissolve and regenerate keratin from chicken feathers,^17^ the referred work serving as motivation and guidance for the current one. The DESs can be obtained by relatively simpler synthesis procedures than the ILs, starting from cheaper raw materials, which could reduce the operational and capital costs of the potential processes based on their use.

**Figure 1:**
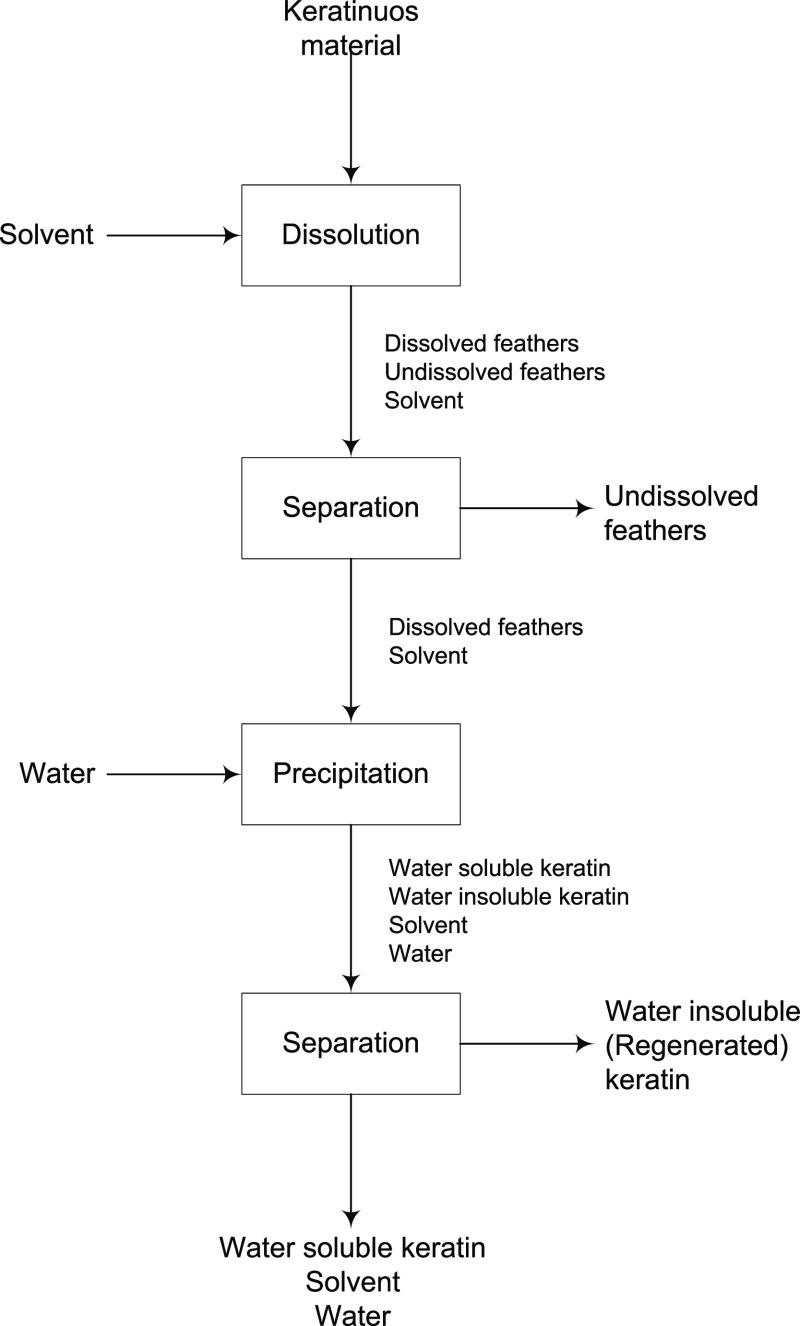
Experimental procedure commonly employed in laboratory experiments for dissolving and regenerating keratin from different keratinous materials by using deep eutectic solvents and ILs (Table S1).

The dissolution and recovery of keratin from chicken feathers (and other keratinous materials as wool, hair, or hoof) with ILs and DESs have been achieved at the laboratory scale using very similar procedures (Figure 1, Table S1), temperature conditions, and reaction times.^17–23,26^

Two fractions of the dissolved keratin (in the DES, for example) are usually recognized by their behavior under water addition: the water-insoluble, also called regenerated keratin, and the water-soluble keratin. Usually (Table S1), large excesses of the solvent, relatively high temperatures (80–180 °C), and reaction times on the order of hours are required to dissolve keratin from chicken feathers. Although solvent-to-feed mass ratios in the interval 6:1 to 50:1 have been explored, the effect of the solvent excess on the solubility of the keratin has not been systematically investigated. In^22^, solvent-to-feed mass ratios between 20:1 and 50:1 were examined, with the finding that the extraction yield of keratin varied in the interval 17.4 to 22.2% approximately with the maximum for the ratio 40:1.

Although the experimental results obtained until now at the laboratory scale^17–23,26^ are promising (Table S1), the rigorous operating conditions required in the process advise an investigation on the technological and economic viability of the corresponding process at larger (i.e., pilot plant, demonstrative, and commercial) scales. Furthermore, the recovery of the DES to be reused in the process, a question that has received scarce attention so far in the studies related to the regeneration of keratin from chicken feathers, demands serious consideration due to the impact it could have on the feasibility of the process proposed, as it has been observed in separation processes based on ILs.^40–42^

To solve the issues described in the previous paragraph, process simulation using commercial program packages is strongly recommended. However, the main components involved in this process are not included in the database of the process simulators. Furthermore, experimental information on the thermodynamic, thermophysic, and transport properties of the individual components and their mixtures is insufficient, which limits both the possibility to create new nondatabank components and to specify the thermodynamic models required for the property’s estimation. By this reason, the use of regressive thermodynamic activity models is unfeasible, with the predictive models such as the conductor-like screening model (COSMO)-based ones being a possible solution. This makes unavoidable the knowledge of the molecular structure of the species involved in the process. The computational procedure used in the current work can be found in the previous papers.^40,43^

In the current work, the techno-economic viability of large-scale processes devoted to dissolve and regenerate the keratin from the chicken feathers using the (NaAc + urea) DES is evaluated by applying Aspen Plus process simulations. This includes the solvent recovery for its reuse in the process. The results obtained previously in laboratory experiments are used as a reference and support for this study. The process simulations are carried out using the COSMOSAC property model implemented in Aspen Properties. Furthermore, an attempt has been made for improving/optimizing the potential industrial process by hypothesizing that the conditions assessed in processes of a similar nature^17–23,26^ can be applied to the current one without incurring limiting inconsistencies.

To fulfill the general objective of this work using a multiscale approach, the structure of the components involved in the process must be established and their properties determined prior to modeling the industrial process. Congruently, the paper is organized in three main parts: (i) the structure of the (NaAc + urea) DES, keratin, and keratin derivatives are established and validated; (ii) the properties of the components involved in the process and their mixtures are predicted and the validity of the predictions examined; and (iii) the mass and energy balances of the process, the equipment sizing, and the process economic analysis are performed.

In summary, the molecular modeling and the process simulations are combined to study different features of laboratory experiments oriented to dissolve and regenerate keratin from chicken feathers using the (NaAc + urea) deep eutectic solvent and to determine the principal aspects of the conceptual and basic engineering of its implementation at higher scales. Accordingly, attention has been paid to the energy consumption, the equipment sizing, and costing of all the processes including the solvent recovery. For the reactor sizing, a kinetic model established from previous laboratory experiments^17^ was used. A process for processing 20 kt feathers/operational year was evaluated. To facilitate the comprehension of the paper, the general fundamentals of the work are explained in the Introduction, while those corresponding to each of its parts are presented in the corresponding sections.

## 2 Computational Details

### 2.1 Molecular Structure

Molecular models of keratin, products of its decomposition, and (NaAc + urea) DES were created by geometry optimization using theoretical methods. The (NaAc + urea) DES, which according to the experimental results^17^ has a NaAc/urea molar ratio of 1:2, was modeled as a cluster composed by one molecule of NaAc and two of urea conveniently disposed to each other. Model keratin-type input structures were proposed after identifying the major amino acids in its composition (Table S2) and their possible ordering. Important simplifications were adopted due to the computational difficulties that the molecular calculations of this type of systems entail. For example, the keratin model included only one S–S bond. Correspondingly, in the products of its cleavage, sulfur participates only in the –SH but not in the S–S bonds. Molecules in the geometry optimization tasks were considered gas-phase-isolated, i.e., not interacting with other molecules or solvent media. Additionally, geometry optimizations were performed steepest using different computational levels: molecular mechanics and semiempirical and density functional theory (DFT) quantum mechanics methods. They were selected depending on the molecular size and complexity of the interactions to be described.

Molecular mechanics and semiempirical geometry optimizations were performed with the universal force field and the Parametrized model 6 (PM6), respectively. Two different DFT computational levels were also utilized. The Becke–Perdew_86 (BP86) functional with def2-SVP basis sets was selected for DFT geometry optimization of the keratin derivatives. m062x functional with polarized and diffused (TZVPD) basis sets were used to optimize the geometry of the DES aggregate for ensuring a good description of the nonlocal interactions prevailing in the cluster constitution. Systematically, frequency calculations were performed on the optimized structures for proving that they correspond to a minimum energy state.

Single-point COSMO calculations^44^ were performed to characterize the electronic structure of the DES and the keratin-type models. In the COSMO calculations, the TZVP basis was used to determine the polarized charge density distribution on the molecular surface of big molecular systems, whereas the TZVPD_FINE basis was used for relatively small molecules or, in those cases, when a rigorous description of the molecular interactions was recommended. The single-point COSMO calculations were done on the corresponding previously optimized geometries.

The molecular mechanics, semiempirical and DFT geometry optimizations, and single-point COSMO calculations were carried out in Gaussian 16.0 and Turbomole 7.3 program packages.

### 2.2 Property Estimation

Conductor-like screening model for real solvents (COSMO-RS) methodology^44^ and quantitative structure–property relationship (QSPR) models based on its descriptors (as implemented in the COSMOtherm program package) were used to predict the thermodynamic, volumetric, and transport properties of the individual components and their mixtures. COSMO-based methodologies have been extensively used in determining the properties of IL, DESs, and their mixtures with conventional organic compounds.^45–50^ The calculated properties received three main uses in this work: (i) characterizing the electronic structure of the components involved in the process. In particular, the density of the polarized charge distribution on the molecular surface was considered, which was reported in the form of the corresponding σ-profile; (ii) studying the interactions among the components in a mixture. For this, the excess enthalpies of the binary mixtures were computed. Moreover, the contributions of the H-bond, van der Waals, and misfit interactions to the excess enthalpies were evaluated; (iii) creating nondatabank components and specifying the COSMOSAC property model in Aspen Plus.

COSMO-RS calculations were performed with the COSMOtherm v 20.0 program package using the BP_TZVP_20 or BP_TZVPD_FINE_20 parametrizations depending, again, on the molecular dimensions of the system considered.

### 2.3 Process Simulation. General Definitions

The components (NaAc + urea) DES, feathers, and both the soluble and the insoluble products of the keratin decomposition were created as pseudocomponents in Aspen Plus. The feathers were modeled as the pseudo-liquid component keratin (KER). The two fractions obtained from the keratin dissolution were named soluble in water keratin (SWK) and insoluble (in water) keratin (ISK). For creating the nondatabank components, the molecular weight, the density, and the normal boiling temperature, calculated by the COSMO-RS methodology, were specified (Table S3). The remainder thermodynamic properties were estimated by the methods and models implemented by default in the Aspen Plus property system.

For validation, the density of the DES (NaAc + urea) calculated in this work (1.3 g/mL) matches well with the densities measured experimentally for DESs composed by tetrabutylammonium bromide and propylene glycol^51^ and the choline chloride-based DESs.^52^ Densities of the same order of magnitude have been measured experimentally and computed by molecular dynamics for the pure glyceline DES,^53^ decreasing with the temperature and with the water content in its mixtures with the later.

The dependence of the viscosity with temperature was specified through the Andradés equation for all the nondatabank components aiming to improve the property estimations. To the authors’ knowledge, experimental information on the η = *f*(*T*) dependence is not available for the DES selected in this work. By this reason, the parameters *A* and *B* (Table S4) corresponding to (tetrabutylammonium bromide + propylene glycol) DES, taken from ref (51), were assigned to the (NaAc + urea) DES. These parameters are in the same order of magnitude as those obtained from experimental measurements of the shear viscosity for the glyceline (choline chloride + glycerol) DES.^53^ In the case of the pseudo-liquids keratin (KER) and its derivatives (SWK and ISK), the *A* and *B* (Table S4) parameters were specified assuming they behave as viscous ILs.^54^

The heat capacity of water was taken from the Aspen Properties database. The models implemented by default in Aspen Plus were used to estimate the heat capacities of the remaining components. The mass heat capacity calculated for the DES (0.962 kJ/kg K) matches well with heat capacities of the ILs (between 0.3 and 1.2 kJ/kg K) obtained experimentally^55^ and calculated by using the same procedure as the one used here.^54^ Unfortunately, also to the authors’ knowledge, no experimental data on the heat capacities of the DESs are available so far. The heat capacities estimated by Aspen Plus for the components ISK and SWK were 2.1 and 4.7 kJ/kg K, respectively. A value of 1.53 kJ/kg K, obtained experimentally for the bovid horns,^56^ was set as the mass heat capacity for the component KER taking into consideration the similarity in composition between this material and the chicken feathers. Furthermore, the sample treatment used in the heat capacity measurement of the bovid horns is like that employed for the feather conditioning in the processes devoted to dissolve and regenerate the keratin from them.

The COSMOSAC property model was selected for estimating the thermodynamic properties of the fluids in the Aspen Plus process simulations. COSMO volumes and sigma-profiles (Table S5) were specified for each component. The conductor-like solvent model–segment activity coefficient (COSMO–SAC) equation^57^ was chosen for estimating the activity coefficients of the individual components in the corresponding mixtures.

A kinetic model of the chemical reaction involved in the keratin dissolution from feathers with the (NaAc + urea) DES was obtained by processing conveniently the results concerning the effects of the reaction time and the temperature on the keratin extraction yield, obtained from ref (17). The fraction of undissolved feathers (undissolved keratin, UKER) was selected as a response function of the model. The kinetic model implementation in Aspen Plus was validated using the batch reactor model available in this program. The batch reactor was specified using the laboratory experimental conditions and residence times.^17^ The KER conversions were calculated and compared with experimental results given in ref (17). The chemical decomposition of the keratin in the presence of the DES was modeled by the stoichiometry given in eq 1.
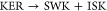
1

**Figure 2:**
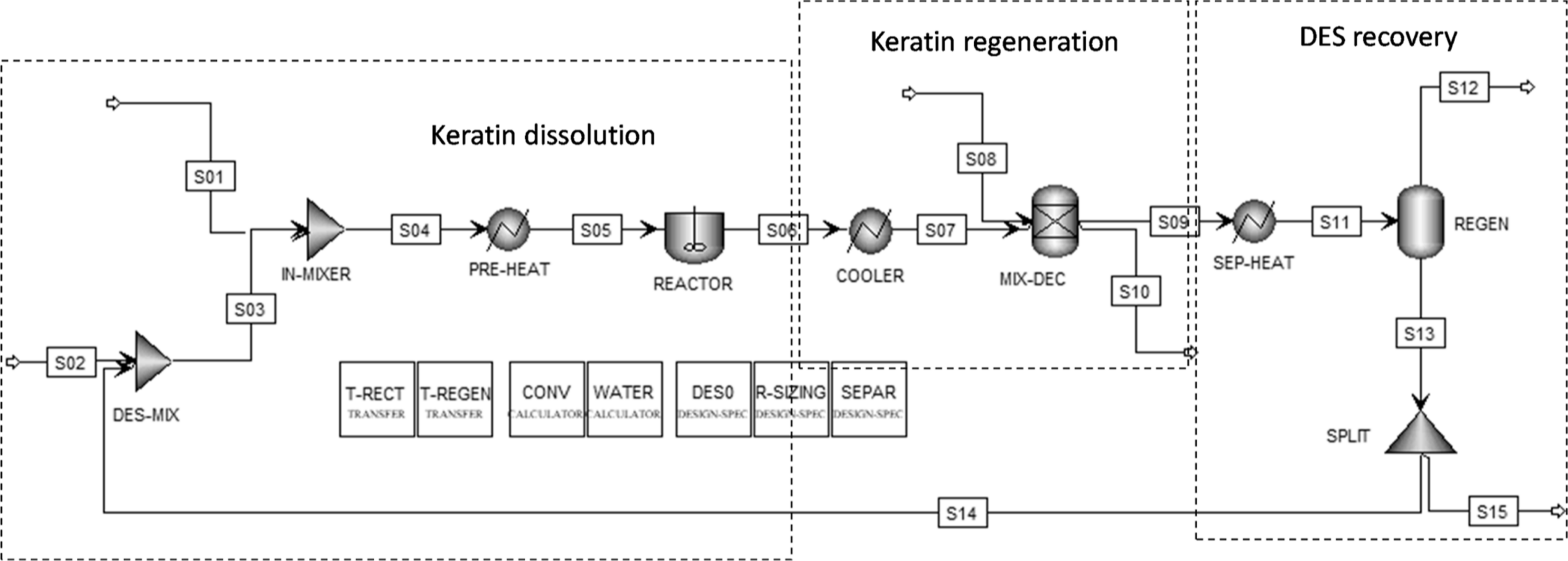
Process model used in Aspen Plus to simulate the keratin dissolution and regeneration process from chicken feathers using (NaAc + urea) DES as the solvent. The process was divided into three sections to facilitate the analysis. They are identified in the flowsheet. This process model is presented only for conceptual design purposes, i.e., it is simplified for clarity. In correspondence, among other simplifications: (i) the heat exchange operations are simulated throughout one-side heat exchangers; (ii) the heating of the water added for keratin regeneration (S08) is omitted; and (iii) the cooling of the streams leaving the process (S10 and S15) to the battery limit’s temperature is also ignored. More complex models are given in Figures S12 and S15.

The process simulations were carried out in Aspen Plus (v14.0) considering a continuous process, the flow diagram of which is shown in Figure 2. The process was divided into three sections to facilitate its analysis. The equipment pressure drops were neglected in all the simulations.

### 2.4 Modeling the Keratin Dissolution

Keratin dissolution is considered here to be the core of the process depicted in Figure 2. Correspondingly, it was first analyzed and optimized individually and then integrated into the entire process.

Streams S01 and S02 represent, respectively, the feathers and fresh DES fed to the process. S02 was considered a (DES + water) mixture containing 90 wt % of the eutectic mixture composed by (NaAc + urea) in the molar ratio 1:2.^17^ Feathers feed flows (S01) of 50–2500 kg/h were considered in an attempt to evaluate pilot plant, demonstrative, and commercial process scales. Results shown in this work are mainly related to the capacity of 20 kt feathers/operational year. The fresh (S02) and the recycled (S14) solvent are mixed (DES-MIX). A mass ratio solvent/feathers (mass flow S03/S01) 50:1 was specified in the base case (Table 1), taking into consideration that the kinetic equation used here was obtained from experiments that employed only this solvent-to-feed ratio.^17^ This value is similar to the one used in several experiments, where ILs have been employed as the reacting solvent (Table S1). Nevertheless, solvent-to-feed mass ratios lower than 50:1 have been evaluated in other experimental studies (Table S1). The Calculator Flowsheeting Option DES0 (Figure 2) fixes the S02 mass flow for each recirculated mass flow (S14) to ensure that the mass ratio S03/S01 is taken as a design specification.

**Table 1:** Process Specifications Considered in the Base and Alternative Cases Explored in the Current Work^a^

Base Case: Defined mainly from the experimental results obtained at the laboratory scale to regenerate keratin from chicken feathers with an aqueous DES (NaAc + urea)^17^

KER_0_ = 2500 kg/h. Solvent-to-feed (S03/S01) mass ratio = 50:1. *T*_S01,S02_ = 25 °C. Single agitated tank reactor operating in isothermal conditions. Reactor operating temperature (*T*_S05_ = *T*_S06_) = 120 °C. KER conversion at the reactor = 60%. Water/mixture (S08/S07) mass ratio = 2.3:1. Operating temperature at the keratin regenerator (MIX-DEC) = 60 °C (*T*_S09,S10_). *T*_S08_ = 60°C (water added is previously preheated). Split fractions to S09 in MIX-DEC: 1.00 for DES and SWK, 0.99 for water, and 0 for ISK and KER. Operating pressure for all the process = 1 bar. Split fraction at SPLITTER = 0 (no recycling of the solvent is considered). Purity of the DES recovered at REGEN = 90 wt % (*T*_S11_ is adjusted to ensure this condition). Flash separator is oriented horizontally. Temperature of the output streams (S10 and S13 in Figure 2) at the battery limits = 40°C. The vapor produced by S12 (Figure 2) was not condensed, instead it was valued as LPS produced by the process. No heat integration was explicitly considered

Alternative Cases: Defined mainly from the laboratory experiments to dissolve and regenerate keratin from different keratinous materials using ILs and DES (summarized in Table S1)

KER_0_: (50–2500) kg/h. Solvent-to-feed (S03/S01) mass ratio = (10:1–50:1). Water/mixture (S08/S07) mass ratio = (0.5:1–2.5:1). *T*_REACTOR_: (80–170)°C. Adiabatic operation at the reactor was also considered. Series of agitated tanks and a tubular reactor were evaluated. *P*_REACTOR_: (1–4) bar. Split fraction at SPLITTER: (0–0.9). No heat integration was explicitly considered

a For nomenclature, see Figure 2. In all the cases, it is considered that the feathers have been conveniently pretreated before to be fed to the process.

The model PRE-HEAT calculates the heat required to increase the temperature of the inlet materials (S04) to the reactor operating temperature (*T*_S05_). The PRE-HEAT outlet temperature is automatically transferred as the specified REACTOR outlet temperature by the transfer manipulator T-REACT when the isothermal regime is selected for reactor operation. A continuous stirred tank reactor (CSTR) model simulates the process reactor (REACTOR). Otherwise, a plug flow reactor model was employed with the same purpose in analyses devoted to improve the keratin dissolution (alternative cases, Table 1).

The kinetic model obtained in this work was used in the reactor calculations. KER conversion according to the reaction shown in eq 1 was calculated by a Fortran code implemented through the calculator CONV (Figure 2). Operating conditions at the reactor in the base case (Table 1) were specified in agreement with previous laboratory experiments.^17^ However, other conditions (alternative cases, Table 1) were investigated for process improvement. The design specification flowsheeting operator R-SIZING (Figure 2) was used to calculate the reaction volume required to reach certain KER conversions/dissolutions for the reaction conditions. A 60% KER conversion was specified for reactor sizing taking into consideration that approximately 60% of disulfide bonds in keratin should be cleaved to be dissolved in ILs.^18^ Series of CSTR reactors were used as an alternative (alternative cases, Table 1) to individual reactors, aiming to reduce capital costs. In these cases, volumes of individual tanks were obtained by minimization of the total volume for certain temperature conditions. The process optimizations were carried out with the optimizer model analysis tool implemented in Aspen Plus.

### 2.5 Simulation of the Keratin Regeneration

In the experiments for dissolving and regenerating keratin from chicken feathers using ILs or DESs (Figure 1), the undissolved keratin is separated from the mixture with the DES and, further, water is added to this mixture. As a result, a certain amount of the products of the keratin decomposition precipitates by the solvent change. These products are considered the regenerated keratin. In the process model created in this work (Figure 2), the mixture leaving the reactor (S06) contains the undissolved keratin (UKER), the products of the keratin decomposition (SWK and ISK), and the solvent. It is cooled (COOLER, S07) and further water (S08) is added, and the separation is carried out in the mixer-decanter (MIX-DEC) equipment model. The MIX-DEC operating temperature was set to 60 °C, and a mass flow ratio S08/S07 of 2.3 was specified in the base case (Table 1) as in the laboratory experiments.^17^ Other water/mixture mass ratios were also proposed (alternative cases, Table 1). The calculator WATER automatically ensures the water mass flow according to the S08/S07 ratio selected. Due to the lack of experimental information to characterize the equilibria involved in this process operation, the MIX-DEC was modeled as a component separator (Sep) in Aspen Plus where the separation is based on specified flows or split fractions by components. For simplicity, both the undissolved feathers (KER) and the regenerated keratins (ISK) are separated together (S10) along with a certain amount of water as humidity. The separation strategy used in the simulations was the following: all the undissolved keratin, the regenerated keratin, and 1% of the total water fed to MIX-DEC were separated through stream S10. The remainder components were conducted (S09) to the solvent regeneration section (Figure 2).

### 2.6 Modeling the Solvent Recovery

The viability of the solvent recovery and reutilization in processes where ILs are used in this role^28^ has been demonstrated in long-term pilot plant experiments.^58^ Furthermore, different process alternatives to recover the ILs have been evaluated^59,60^ and the impact of their regeneration on the design and economy of the separation processes has been evaluated.^40^ This question has also been addressed in laboratory experiments to dissolve and regenerate keratin from chicken feathers with ILs.^21,22^ The recovery and reuse of DES have been less investigated; however, the results obtained up to now^61–64^ allow accepting the possibility of doing so, regardless of further research on economical and efficient regeneration methods for DES being required.^63^

In the base case (Table 1) of the current process proposal, the DES must be recovered from a mixture (S09) that contains approximately 70 wt % of water and around 1 wt % of water-soluble products of the keratin decomposition (which are also soluble in the DES, Figure 1). The water could be removed by vaporization, but the products of the keratin decomposition require another technique to be eliminated due to their high boiling temperatures. A two-outlet flash separator (Flash2) has been used to model the solvent regenerator (REGEN). The mixture (S09) is previously heated in the SEP-HEAT. The heating temperature (T_S11_) is adjusted by the Design Specs SEPAR, so the recovered solvent (S13) has 90 wt % of the (NaAc + urea) DES for the pressure specified. Afterward, the outlet temperature of SEP-HEAT is transferred by the transfer manipulator T-REGEN as the operating temperature of REGEN. The bottom product (S13) of the distillation column is divided (SPLIT) in two fractions: the first one is recycled to the process (S14) and the second one (S15) is sent to another unit (not considered in this work) for eliminating the products of the keratin decomposition dissolved in the DES as recommended, for example, in refs (61) and (63) This fraction can be fed as a fresh solvent (S02) after purification. Several split fraction scenarios (alternative cases, Table 1) were evaluated taking into consideration that the recycled solvent could have different capacities to dissolve keratin from chicken feathers. The solvent recovery and recycling state the problem of the process integration as reflected in Figures 2, S12, and S15.

### 2.7 Base Case and Process Improvements

The performance of the process shown in Figure 2 was assessed for a set of base specifications (Table 1, base case), which were selected according to the following two criteria: (i) to reproduce, as closely as possible, those used in the laboratory experiments taken as reference for the current work;^17^ (ii) a process capacity of 20,000 t/year was selected for considering commercial scales.

In addition, alternative cases were proposed and analyzed aiming at process optimization. To select the operating conditions in these cases (Table 1), the results of other works on the keratin dissolution and regeneration from different raw materials with ILs (Table S1) were considered.

It is important to recognize that the conclusions derived from the alternative cases should be taken with care for the following reasons: (i) reaction temperatures and solvent/feed ratios different from those experimentally proved could affect the reaction kinetics; (ii) keratin regeneration has been scarcely investigated, and themes such as the effect of the water added on its efficiency have not yet been solved. Here, it is considered that the S08/S07 mass ratios specified do not affect the keratin regeneration but only the energy consumption and equipment sizes. Thus, the accuracy of some results corresponding to the alternative cases is consistent with the conceptual engineering level in process developments. Anyway, as already acknowledged in the Introduction, it can be accepted that conditions assessed in processes of a similar nature (Table S1) can be applied to the current one without making mistakes that affect the essence of the process proposal presented here.

### 2.8 Equipment Sizing and Cost Estimation

The main equipment resulting from the basic design of the process (Figures 2 and S12) was sized and costed. The reaction volumes obtained from process simulations by the Design Specs R-SIZING were converted to reactor volumes considering that the tank was oriented vertically, and the liquid percent level was 85%. The ratio length-to-internal diameter of the reactor was assumed to be 3:1. The REGEN was sized as a horizontal LV-separator due to economic reasons^65^ using the Vessel Sizing Equipment Design utility available in Aspen HYSYS (v 14.0). For this, the Property System created in Aspen Plus was fully transferred as a Fluid Package to Aspen HYSYS. The heat exchangers were sized by the Aspen Exchanger Design and Rating (Aspen EDR, v 14.0) after wholly exporting the simulation results from Aspen Plus to Aspen EDR. Due to the lack of information on the keratin regeneration equilibrium, the MIX-DEC was roughly sized by the equation *V* = *t*_Res_·*Q*_V_, where *V* is the volume, *t*_Res_ is the residence time of the liquid (20 min), and *Q*_V_ is the volumetric caudal fed to the vessel, which was estimated as *Q*_V_ = *Q*_V(S07)_ + *Q*_V(S08)_.Vessels wall thicknesses of 8.1 and 9.7 mm were assumed because they are the minimum values necessary to maintain the structure integrity of vessels having internal diameters in the ranges 1.07–1.52 m and over 1.52 m, respectively.^65^ When the process proceeded (vessels, tubes at the heat exchangers, etc.), stainless-steel SS-304 was selected as the material of construction considering the potential corrosive and erosive character of the mixtures. Equipment costs were determined by using the Aspen Capital Cost Estimator (ACCE, v 14.0). Details of the equipment selection (mapping) for the cost estimation are given when appropriate.

Saturated steam was selected as the heating fluid in those operations where required. Low-pressure steam, medium-pressure steam, and high-pressure steam were selected, respectively, for operating temperatures lower than 100 °C, up to 120 °C, and higher than 120 °C. Water proceeding from a cooling tower (*T*_in_ = 35 °C, *T*_out_ = 50 °C) was used as the cooling fluid. Electric drivers were selected for operations where it was necessary, such as the pumping of the fluid. SS-304, LPS, MPS, HPS, and cooling water were quoted at 16.35 $/kg, 1.9 × 10^–6^ $/kJ, 2.2 × 10^–6^ $/kJ, 2.5 × 10^–6^ $/kJ, and 2.25 × 10^–7^ $/kJ, respectively. Electricity was quoted at 1.58 × 10^–5^ $/kJ. Prices of the stainless steel and the process utilities were taken from the Aspen Economics v 14.0 databank and correspond to the first quarter of 2022.

Total annual costs were determined as the sum of the annuities derived from the purchasing equipment costs and utilities costs. For annuity estimation, it was considered that the equipment costs were refunded in 10 years, accepting a refund ratio constant in all this period. More details on the equipment sizing and costing are given in the Supporting Information.

## 3 Results and Discussion

### 3.1 (NaAc + Urea) Deep Eutectic Solvent: Structure and Properties

**Figure 3:**
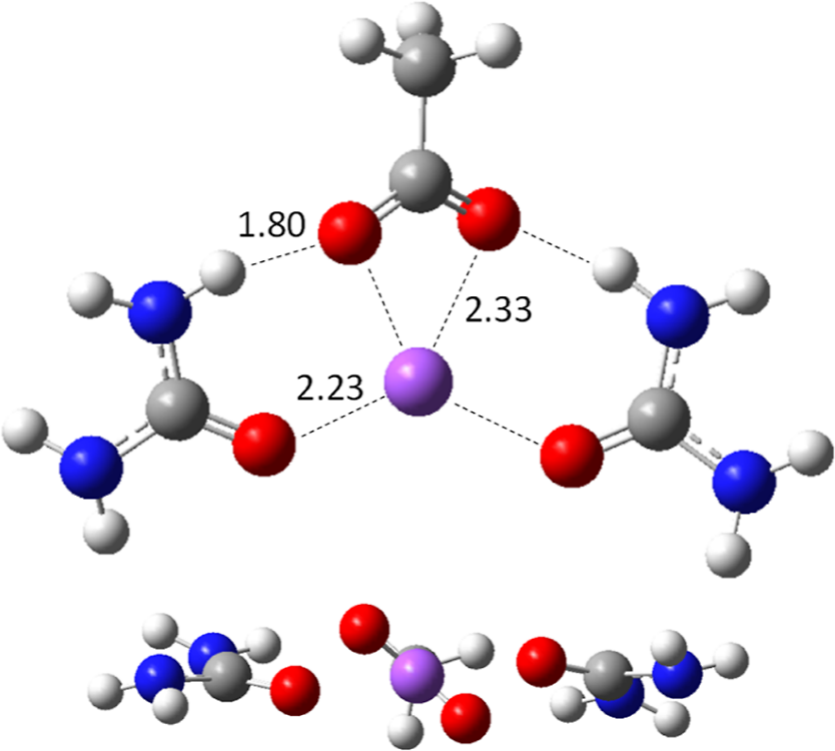
Optimized gas phase structure of the NaAc/urea (1:2 molar ratio) cluster governed by the H-bond interactions (Experiment S1). m06-2*x*/6-311G(d,p) calculations. Superior view [up]. Lateral view [down]. Atom color code: violet, sodium; blue, nitrogen; red, oxygen; gray, carbons; light gray, hydrogen.

Figure 3 shows the structure obtained considering the gas-phase isolated cluster, i.e., not interacting with other molecules or solvent media. It is quasi-planar, with the urea molecules interacting simultaneously with sodium cations and oxygen atoms of the acetate anion. Interatomic H···O distances of 1.8 Å are typical of H-bond interactions, which play a significant role in the molecular packing (Experiment S1). Na···O interatomic distances in NaAc increases 5.6% respect to the individual molecule because of the interaction with urea molecules. Similar structures have been reported for other DESs^32,52,53,61,66^ having approximately the same composition.

Using the COSMO-RS methodology to estimate the thermodynamic properties of the DES and its mixtures forces us to define how to model this component. The two alternatives more frequently considered in the literature^45–48^ with this purpose were explored here: (i) representing the (NaAc + urea) DES as a mixture of the individual components (in the current case 1NaAc and 2urea molecules) or (ii) considering the DES is the molecular aggregate shown in Figure 3.

**Figure 4:**
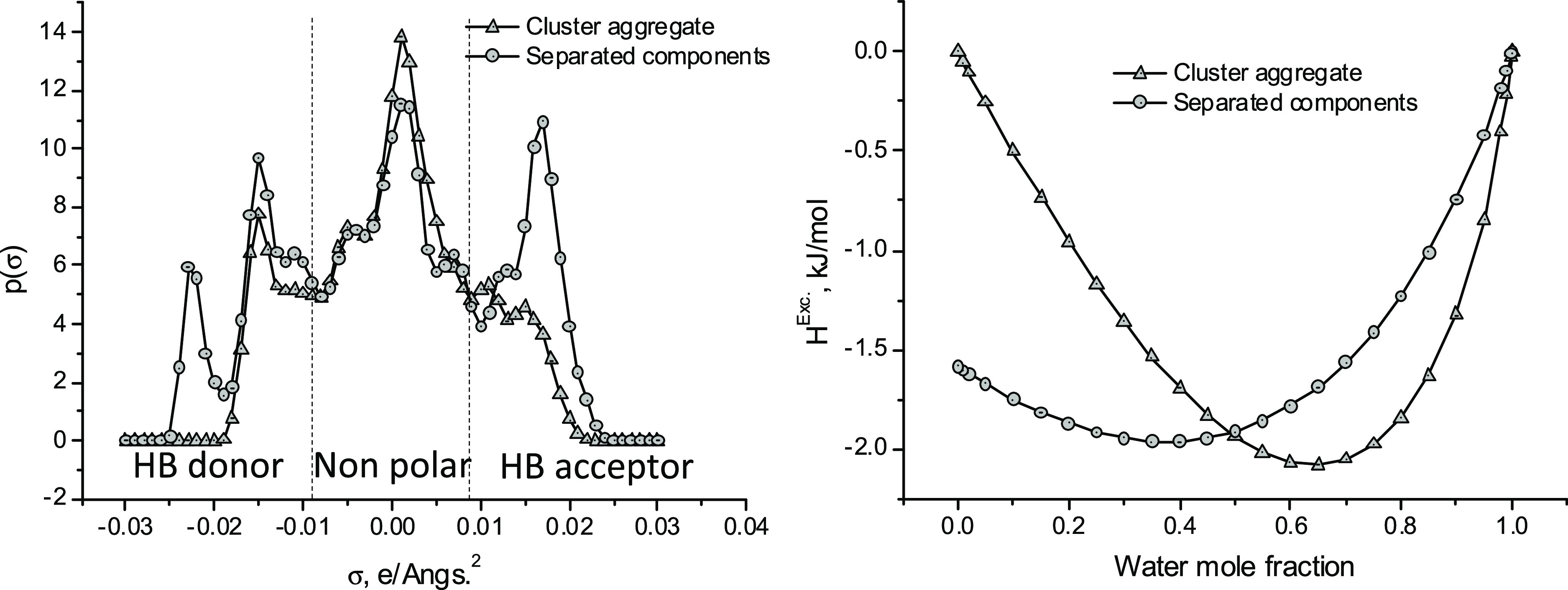
Sigma-profiles (σ-profiles) obtained by COSMO calculations for the DES NaAc/urea (1:2 molar ratio) considering that the solvent is a mixture of the individual components or, alternatively, a molecular aggregate (Figure 3) [left]. Excess enthalpies of mixtures (DES + water) for both structural models of the DES. COSMO-RS calculation. *T* = 298 K. [Right].

For the DES selected in this work, the formalism of the separated components overestimates the polarity of the solvent as indicating the presence of intense peaks in its σ-profile at approximately σ = +0.02 e/Å^2^ and σ = −0.02 e/Å^2^ (Figure 4).

They are related, respectively, to the highly polarized carboxylic oxygen atoms and sodium of the NaAc. In the cluster structure depicted in Figure 3, the intramolecular interactions described above significantly reduce the capacity of the complex to interact with other molecules, which is reflected in its σ-profile by the drastic reduction in the intensity of intense peaks shown at approximately σ = +0.02 e/Å^2^ and σ = −0.02 e/Å^2^ (Figure 4).

This fact strongly modulates the properties of the DES such as, for example, its solvent capacity in processes where it is used as such or its ability to interact with conventional molecular species. Thus, excess enthalpies of mixtures (DES + water) significantly differ from one case to the other (Figure 4). This picture could be generalized for any other property of the DES. In correspondence, in this work, the (NaAc + water) DES was modeled as the aggregate shown in Figure 3. Furthermore, it was considered that the cluster retained the structure unaltered for any process condition, i.e., no dissociation of the aggregate occurs.

In the laboratory experiments of keratin extraction from chicken feathers with (NaAc + urea) DES,^17^ 10 wt % of water was added to the solvent. This decision was justified by the water capacity for reducing both the lattice energy of the mixture through new hydrogen bonds and the solution viscosity without destroying the H-bonds between the DEŚs components. To rationalize this decision, in the current work: (i) the structure of the (NaAc + urea) cluster was optimized considering that the aggregate interacted with a solvating medium, whose dielectric constant is that of water. This is a continuum solvation model calculation,^67^ supported by the polarizable continuum model (PCM) using the integral equation formalism as implemented in Gaussian 16.0 and (ii) the viscosity of the pure components and the (DES + water) binary mixtures were estimated by the QSPR formalism available in the COSMOtherm program with this purpose.

Results of the PCM geometry optimization considering water as solvent (Figure S1) demonstrated that the (NaAC + urea) DES cluster structure resulted in somewhat relaxed respect to that of the gas phase structure (Figure 3) due to the interaction with water but without affecting the general molecular arrangement. H···O and Na···O interatomic distances increase 3.9 and 1.1%, respectively, with respect to the gas phase structure.

On the other hand, viscosities calculated by the QSPR model supported by the COSMO descriptors (available in the COSMOtherm program package) showed that the (NaAc + urea) DES is much more viscous than water as already demonstrated experimentally for other DESs.^47,51–53,68,69^ However, its viscosity is reduced 55% in the mixture containing 10 wt % water (supposing that the viscosity of the mixture obeys an ideal mixture rule). These results agree with experimental and computational studies in mixtures of water with choline-based ILs and their derivatives,^52,53,68^ where it was observed that microscopic intraionic interactions within aggregates are not substantially changed until the water content exceeds a 0.65 mole fraction. Furthermore, the viscosity of the ILs and DES was reduced up to 85% with water contents lower than 15 mol %.

The undesirable high viscosity of the (NaAc + urea) DES selected in the experiments carried out by^17^ was additionally mitigated by the relatively high operating temperature of the process. Experimental studies and theoretical calculations^47,51,69^ have demonstrated that the viscosity of pure DESs could decrease up to 80–85% when temperature increases from 25 °C to approximately 90–95 °C.

### 3.2 Modeling the Components of Keratin and the Products of Its Transformation

As already indicated in the Introduction, modeling both keratin and the products of its transformation is a challenge and a key task for the current work. Keratin is a protein with molecular weight over 50 kDa, inaccessible for most of the models and computational resources employed in the multiscale approach used in this work.^43^ Moreover, few crystallographic data have been collected for this protein up to now. In^9^ was published a molecular dynamic optimized structure of keratin. Due to its molecular dimensions (153,379 atoms and 598,302 electrons), several difficulties may arise if the mentioned structure is tried to be manipulated aiming to obtain a computationally viable model for the purposes of this work. Based on these facts, creating keratin molecular models of lower dimensions seems to be the best option.

The individual cysteine amino acid, the oxidized glutathione (GSSG, Figure S2), and a polypeptide composed by two cysteine groups and other amino acids (with a molecular weight of ca. 835) have been used to model the keratin in experimental and computational research on keratin dissolution from wool and feathers with ILs.^18,70^ This way, it was demonstrated that (i) both the S–S bond cleavage rate and the dissolution efficiency are related to the distribution of the IL around the cysteine group and their interactions^18^ and (ii) keratin dissolution in ILs is governed by H-bond interactions. Remarkably, the results given in ref (70) show that the capacity to model keratin in dissolution processes increases as the molecular size of the model increases, i.e., the oxidized glutathione and the polypeptide represent keratin dissolution better than cysteine. However, the dimensions of these structures were yet insufficient to model the keratin regeneration from the DES solutions by adding water (Figure 1). Indeed, the COSMO-RS calculations (Figure S3) suggest that the solubility of GSSG in water is thermodynamically favorable for all of the composition intervals of the mixtures (GSSG + water).

Here, more robust structural models of keratin and the products of its decomposition are proposed, aiming to gain a better description of their interactions with the solvents (DES and water) in the process under study (Figure 1). The alternative proved here consists of creating relatively high-molecular-weight (>1400 g/mol) structures able to reproduce qualitatively the main properties of these components in the process studied. To do so, two questions must be solved: (i) which amino acids should be included in the model structures and (ii) how to order the amino acids in the models.

First, the amino acid composition of the bird’s feather keratin reported in the literature was analyzed, and the most abundant amino acids were determined (Table S2). Indeed, keratin contains about 20 amino acids, but some of them are in the minority (Table S2) and could be omitted during the construction of the protein molecular model. The amino acid composition of the birds’ feather keratin reported by different authors (Table S2) is quite similar regardless of the experimental technique used in its determination, the bird species, etc. Interestingly, half of the amino acids (e.g., serine, glycine, proline, valine, leucine, glutamic acid, cysteine, alanine, aspartic acid, and arginine) constitute approximately 80 mol % of keratin’s composition. On this base, a keratin model with the amino acid composition (1) was proposed
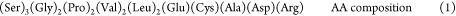
where positively (Arg) and negatively (Asp, Glu) charged amino acids are present as well as hydrophobic (Ala, Cys, Val, Leu), hydrophilic (Ser), and conformationally special (Pro, Gly) ones.

Afterward, the amino acids’ ordering for the keratin model with AA composition was generated considering the protein sequencing proposed by^7^ for the keratin feather of fowl. Thus, amino acids contained in the model fragment (AA 1) were arranged by order of appearance in the sequence reported by.^7^ Interestingly, among the most frequent amino acids in the feathers’ composition [AA composition (1)] are included two negatively charged ones (Asp and Glu) but only one positive (Arg) one. For preserving the electrical neutrality, two molecular models were proposed (Frag. 1 and 2): one containing Glu (Frag. 1) and a second with Asp (Frag. 2).
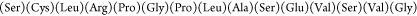
F1
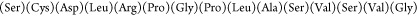
F2

Fragments F1 and F2 could represent the products of the disulfide bond cleavage during the chicken feather dissolution.

Preliminary (input) geometries of fragments F1 and F2 were obtained by using the amino acid sequencing tool available in the program HyperChem (v 7.0) (Figure S4). Both structures were characterized by a linear-shaped moderately folded configuration mainly due to the conformationally special amino acids. Moreover, a model of keratin was obtained joining both fragments through the sulfur atoms (Figure S5). Next, the molecular structures of the keratin fragments and the keratin models were optimized. Geometry optimization of such large and complex structures (Table S6) is somewhat difficult. This complexity is increased by the presence of the already mentioned conformationally special amino acids in the structures, which could lead to several equivalent conformations. To avoid these issues as much as possible, the computational strategy described in the section Computational Details was followed. Geometry optimization of fragments F1 and F2 was sequentially performed at molecular mechanics, semiempirical, and DFT computational levels. The keratin model structure was optimized at the molecular mechanics level.

**Figure 5:**
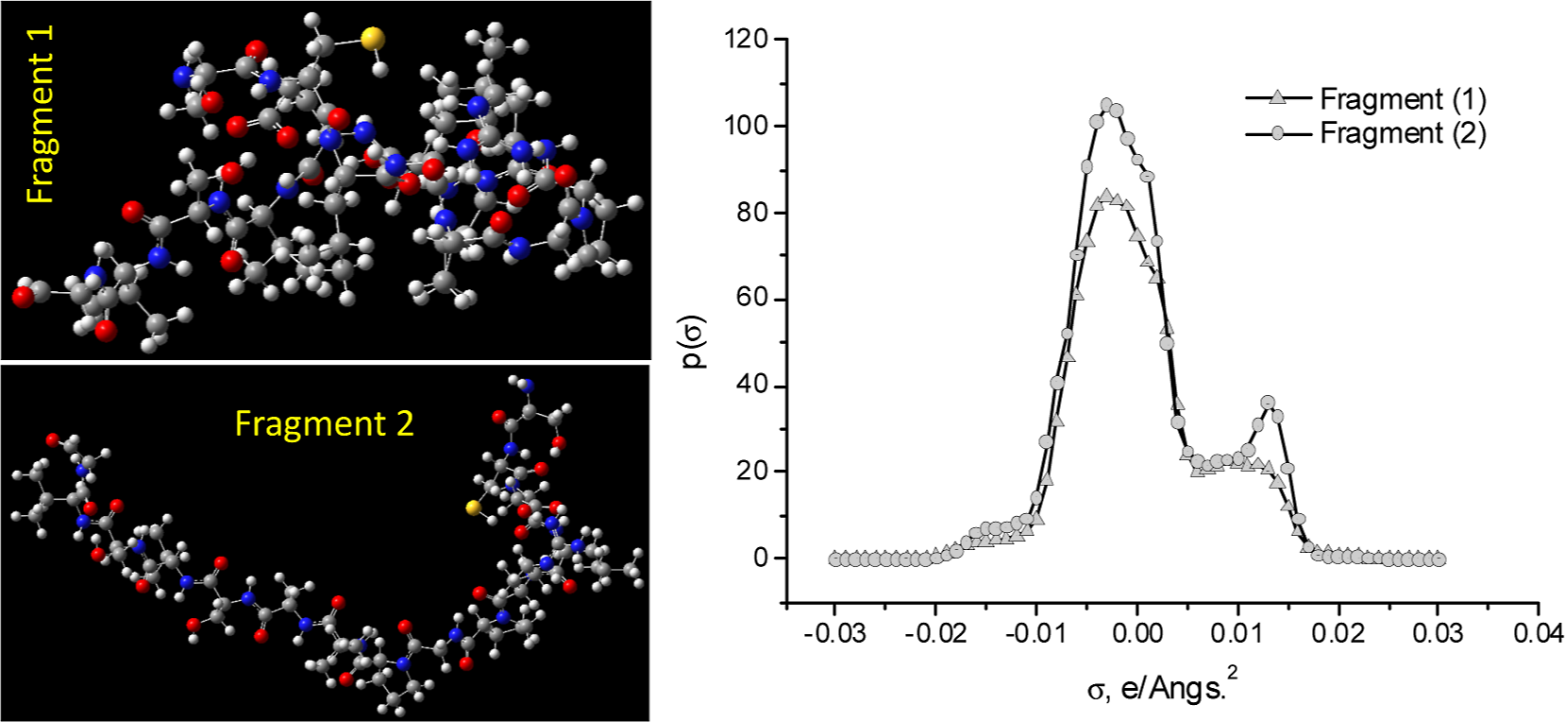
Optimized geometries of keratin fragment models (1) and (2) obtained at the BP86/def2-SVP computational level starting from the input geometries shown in Figure S4. This figure is shown for guidance purposes because the complex 3D packing of the structure hinders its vision in two dimensions [left]. Sigma-profiles of the molecular fragments F1 and F2 proposed in the current work to model the products of the keratin decomposition by DES action. COSMOtherm calculation [right].

The optimized geometries (Figure 5) of fragments F1 and F2 significantly differ from each other. Fragment F2 essentially retained the initial structure, whereas fragment F1 experienced a severe distortion, adopting a crowded configuration resembling a ball. This result shows that small changes in the amino acid composition or its sequence can lead to important changes in the molecular electronic structure of proteins (Table S7, Figures 5 and S6). It is interesting to note (Figure 5, Table S7) that fragment F2 has more active hydrogen bond acceptor groups (peak at σ ∼ 0.015 e/Å^2^) but, simultaneously, more hydrophobic character (peak in the region between approximately −0.01 and 0.01 e/Å^2^) than fragment F1.

**Figure 6:**
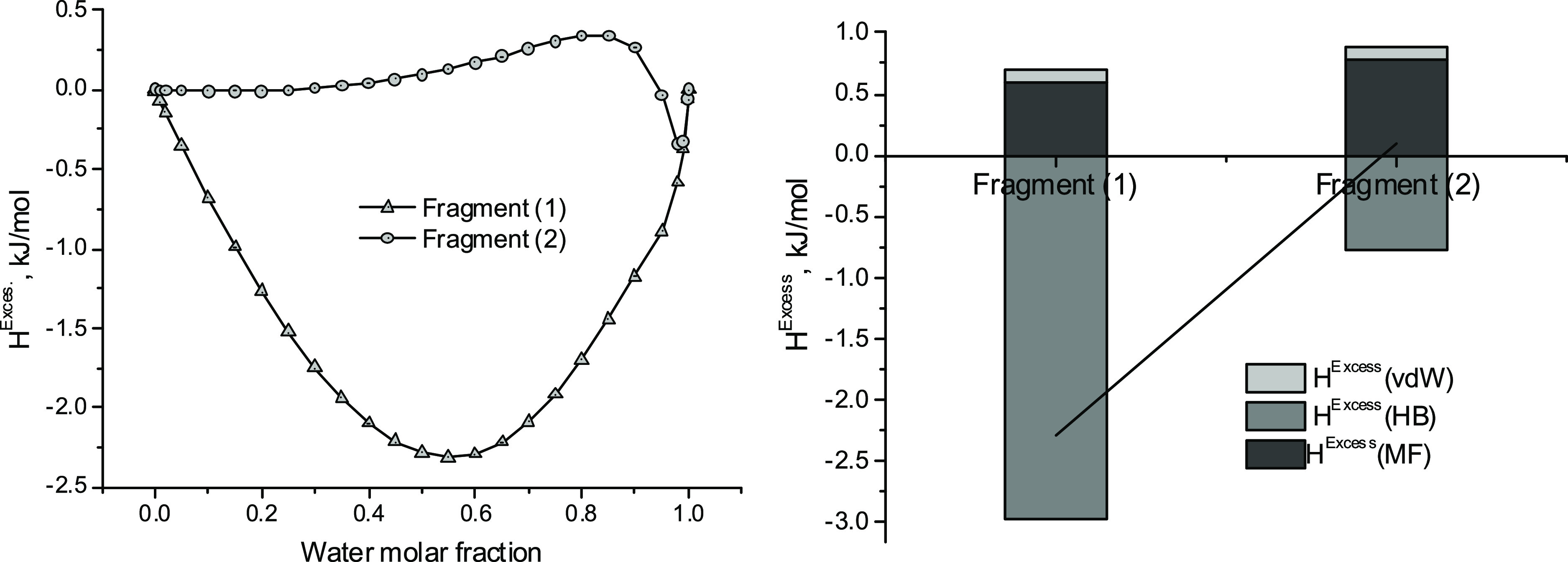
Excess enthalpies for the mixtures (keratin fragments + water) [left]. Contribution of van der Waals, H-bond, and Misfit interactions to *H*^Excess^ in an equimolar mixture of both components [right]. Values on the line correspond to *H*^Excess^ for such a composition. COSMO-RS calculations. *T* = 298 K.

The thermodynamic properties of mixtures (keratin fragment + water) also differ markedly between both keratin fragments (Figure 6). The keratin fragments F1 and F2 interact rather differently with water as resulted from the *H*^Excess^ performance (Figure 6). In both cases, H-bond interactions are favorable to the solubility of keratin fragments in water; however, they are more intense for keratin fragment F1. van der Waals and Misfit interactions behave hydrophobically in both systems.

These results agree with previous experimental observations,^12,13^ where it was found that crowded forms of keratin (like fragment F1) are more soluble than the more extended ones (such as fragment F2). On the other hand, changes from the hydrophobic to hydrophilic character of the keratin have been also described in pretreatments of the chicken feathers for several applications.^1,2,22,24^

The previous results allow us to propose the keratin fragment F1 as a model of the soluble keratin (SWK) in the dissolution processes of this study. Otherwise, the keratin fragment F2 could represent the insoluble (regenerated from an aqueous medium) keratin (ISK). This decision implies that all the S–S bonds present in the raw keratin are broken during the extraction process, as derived from the fact that the sulfur in the products of the keratin extraction (fragments F1 and F2) are taking part of –SH bonds (Figures 5 and S4). This is not real and suggests the necessity of more robust modeling of these systems if a quantitative description of their behavior is desired. The presence of disulfide bonds still in regenerated keratin has been demonstrated experimentally.^26^

The COSMO-RS calculations predict changes with temperature in the solubility and the thermodynamic behavior of the keratin fragments in water (Figure S6). This is relevant in processes of keratin dissolution and regeneration with DESs and other solvents because they are performed at relatively elevated temperatures (Table S1). These changes (Figure S6) are mainly related to the weakening of the H-bond interaction between keratin fragments and water as the temperature increases (Figure S7). The results shown in Figure S7 differ from experimental observations, which demonstrated that a higher dissolving temperature can accelerate keratin degradation. According to,^18^ it is associated with the rupture of the α-helix structure and disulfide bond breakage. The disagreement outlined here suggests, again, the necessity of a more robust molecular modeling of these systems. A reasonable solution to this issue is creating a set of individual structures of different molecular weights to represent the products’ diversity during the keratin decomposition. However, such solutions should be taken with carefulness because they demand large computational efforts.

**Figure 7:**
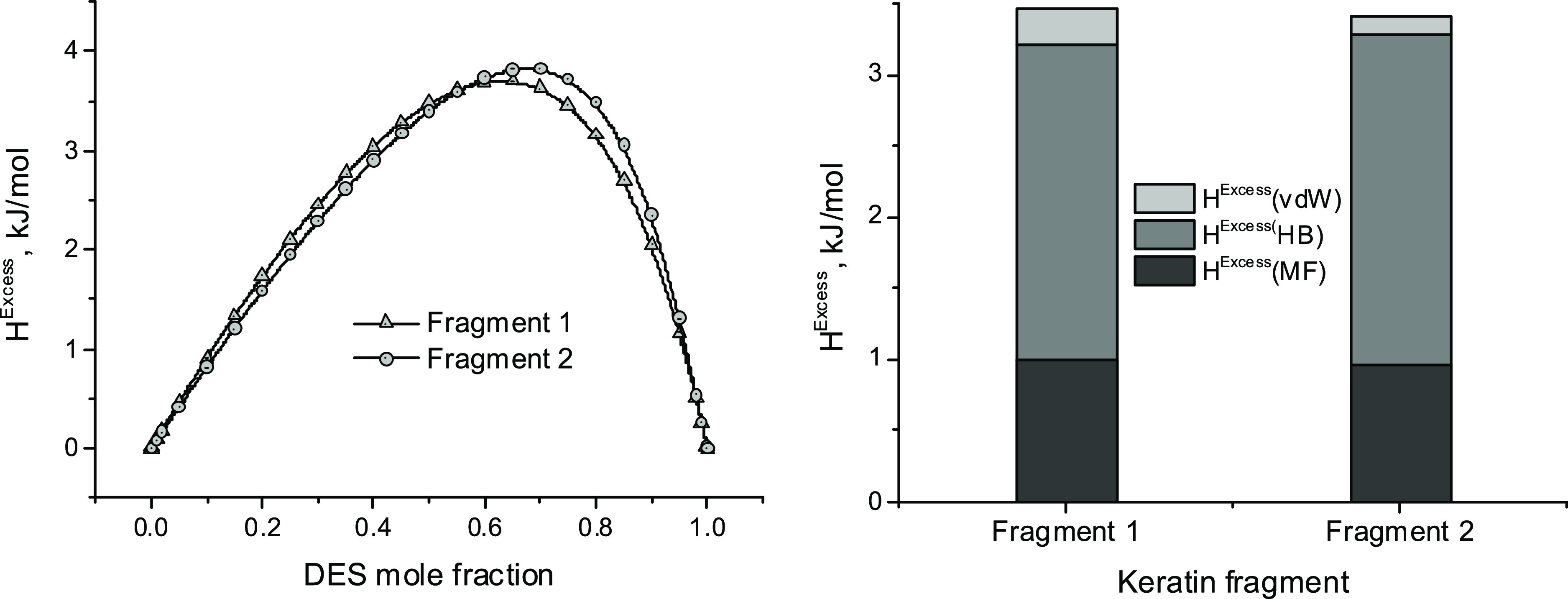
Excess free energy and enthalpy for mixtures (keratin fragments + DES) [left]. Contribution of van der Waals, H-bond, and Misfit interactions to *H*^Excess^ in an equimolar mixture of both components [right]. COSMO-RS calculations. *T* = 298 K.

The molecular models proposed in the current work to represent the keratin decomposition products succeed also in describing changes in thermodynamic behavior when different solvents (water or DES) are used. This fact is evident if Figures 6 and 7 are compared.

It is relevant to note that the interactions of both keratin fragments with the selected DES (Figure 7) are similar but differ when water (Figure 6) is selected as a solvent. This agrees with the experimental procedure used in processes to dissolve and regenerate keratin from feathers by using ILs and DESs (Figure 1). In mixtures (keratin fragment + DES), van der Waals and misfit interactions preserve (with respect to the mixtures with water) the hydrophobic character but H-bond interactions turn out to be nonfavorable (Figure 7).

### 3.3 Kinetic Model of the Reaction Associated to Keratin Dissolution

The results of the laboratory experiments carried out by^17^ show a linear dependency (with *R*^2^ = 0.953) in the form  vs *t* (Figure S8), where UKER and KER_0_ represent, respectively, the undissolved feathers for each time and the feathers fed to the process. This indicates that the dissolution of keratin from feathers with the (NaAc + urea) DES can be represented by a kinetic pseudo-first-order equation. The kinetic eq 2 was adopted in the present calculations.
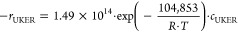
2where *r*_UKER._ is the rate of undissolved keratin disappearance (kmol/m^3^ h). *R* is the gas constant. *T* is the absolute temperature. Reaction time is given in hours. *c* is the molar concentration in kmol/m^3^.

The estimated activation energy of 104.9 kJ/mol (Figure S8) for the dissolution of keratin using the (NaAc + urea) DES is quite high compared to conventional chemical reactions. It is related to the large residence times observed in the laboratory experiments.^17^

The kinetic model (eq 2) was implemented in Aspen Plus and validated by calculating the fraction of undissolved feathers for different reaction times and temperatures assisted by the batch reactor model. A mean relative error of 10.1% was obtained when computed data were compared with experimental data (Figure S9).

### 3.4 Heat Requirements and Equipment Sizing for Keratin Dissolution

The analysis accomplished in this paragraph is related exclusively to the keratin dissolution section of the process proposed (Figure 2). The results shown were obtained considering that all the solvent fed to this section was fresh (at 25 °C); i.e., the recovered solvent was not recycled. Thus, the heat requirements calculated represent the maximum heat consumptions for preheating the raw materials.

The heat necessities and the equipment size depend on both the mass of feathers treated (process scale) and the operating temperature at the reactor, the dependencies being clearly nonlinear with respect to temperature and opposite each other (Figure S10, Tables S8 and S9). The heat duty calculated for the highest biomass charges (2500 kg KER/h) and the highest operating temperature examined experimentally in the reactor (120 °C) is about 4.8 MW, which represents a cost, to this unique concept, of approximately 306.7 × 10^3^ $/operating year for the economic scenario considered in the present calculations. The preheating operation requires a heat exchange surface area of approximately 220 m^2^, the corresponding annuity being 31.5 × 10^3^ $/year for the economic conditions defined. On the other hand, a reactor volume of approximately 5055 m^3^ was calculated for the highest material charges and lowest reaction temperature (80 °C), which entails annuities of about 925 × 10^3^ $/operating year according to the current economic conditions. A minimum total annual cost of approximately 420 × 10^3^ $/operating year was determined for *T* ≈ 120 °C (Figure S11) considering that the reactor (stirred tank) operates isothermally. The economic analysis in this optimization was limited to the steam consumed in PRE-HEAT as well as the REACTOR and the PRE-HEAT purchase costs.

According to the calculations performed in this work, the chemical reaction under consideration is exothermic. The reactor was considered isothermal in the calculations of the preceding results (Figures S10 and S11 and Table S9). Otherwise, if the reactor operates in adiabatic mode, it allows taking advantage of the heat released by the chemical reaction to increase its velocity. This reduces the reactor size without increasing the heat consumption for the thermal conditioning of the inlet materials.

**Figure 8:**
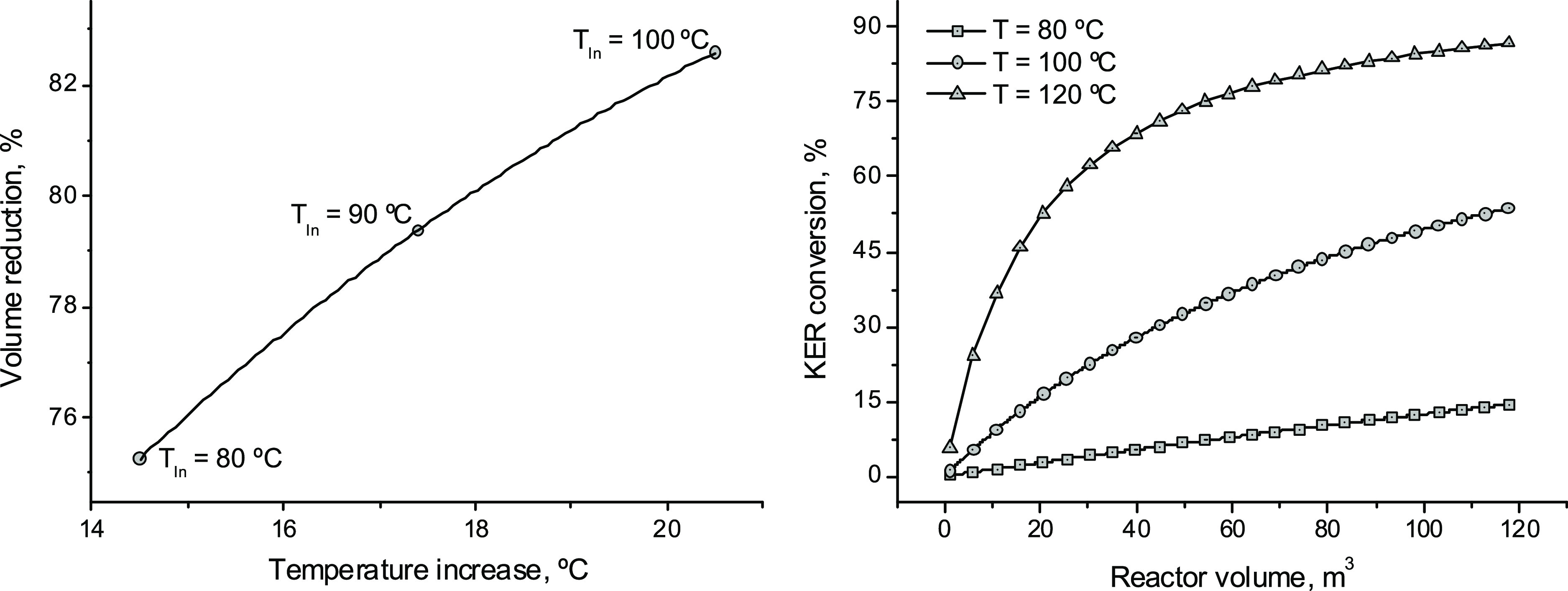
Influence of the adiabatic regime of operation for the reactor of Figures S10 and S11 on its sizing. In the *x*-axis is plotted the increase of reaction temperature under adiabatic operation. In the *y*-axis is the reduction in the reaction volume required to meet 60% KER conversion with respect to the isothermal operation [left]. Dependence of the KER conversion with reaction volume at different reaction temperatures [right]. A single stirred tank reactor was considered. The remainder specifications correspond to the base case (Table 1).

Reduction of the reactor volume correlates well with the temperature increase (Figure 8), when the reactor operates in the adiabatic regime for similar conditions to those of Figures S10 and S11. On the other hand, KER conversion increases with reaction volume as expected (Figure 8) but exhibits asymptotic behavior for the higher temperatures considered in the experiments.^17^ The asymptotic character of this dependence suggests that a series of reaction tanks could reduce the total reaction volume required to reach a certain conversion (Table S10). Reductions of 21.5 and 27.5% in the total reaction volume were obtained when the overall 60% KER conversion was reached in two and three tanks, respectively, disposed in series (Table S10). The use of a series of tanks allows the operation of individual reactors under different thermal regimes, which opens multiple optimization alternatives.

Theoretically, an infinite number of stirred tank reactors are equivalent to a tubular reactor. Adopting this configuration for the reaction unit, the reactor volume required to reach 60% KER conversion is reduced almost 50% at *T* = 120 °C with respect to the situation where a single stirred tank is considered (Table S10).

**Figure 9:**
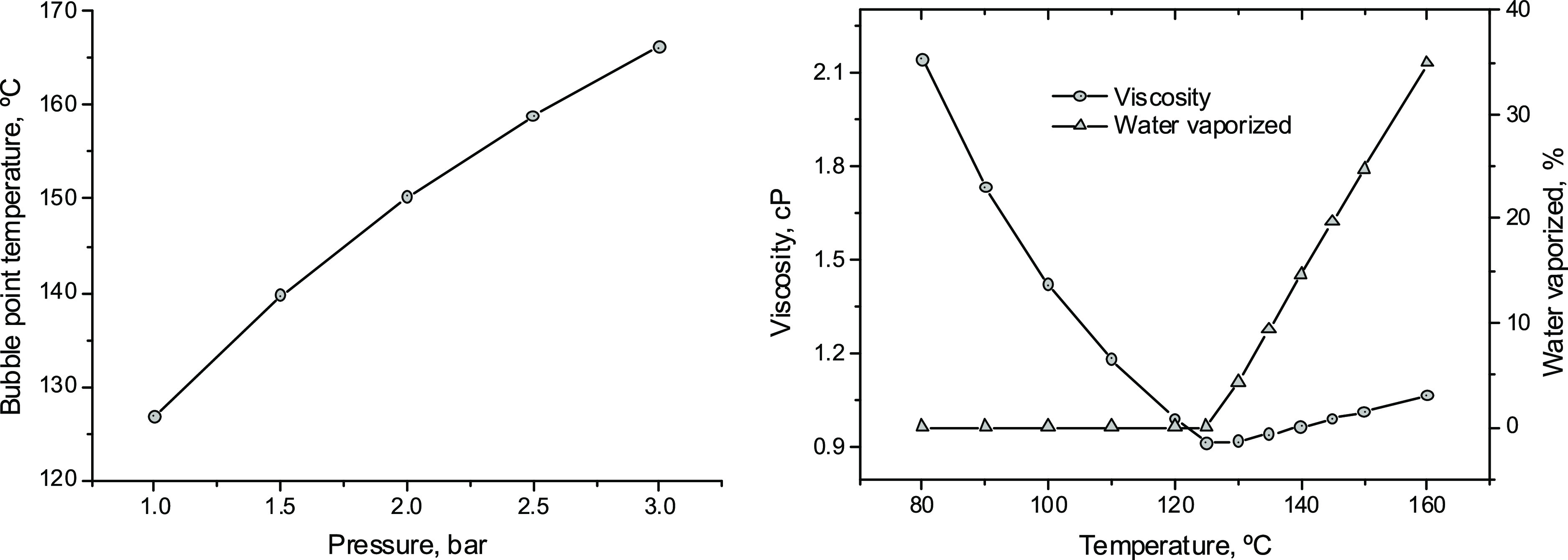
Bubble point temperature of the reacting mixture as a function of the pressure [left]. Water vaporization and viscosity of the reacting mixture for different temperatures at atmospheric pressure [right].

As already discussed, the temperature increase increases the reaction velocity and additionally reduces the viscosity of the reacting mixture, which favors mass and heat transfer phenomena. This has a beneficial effect on the energy consumption associated with the agitation of the mixture. However, temperature increase should be controlled because water added to the DES can be vaporized (Figure 9).

COSMOSAC property model in Aspen Plus predicts that water vaporization begins at approximately 126 °C for the reaction mixture at atmospheric pressure. The discrete increase of the mixture viscosity calculated for temperatures higher than this one seems to be a direct consequence of the water vaporization (Figure 9). A potential solution to this problem consists of operating at pressures over the atmospheric one. A moderate increase in the pressure up to 5 bar ensures reaction temperatures of about 170 °C without water vaporization. For example, operating at *P* = 4 bar and *T* = 170 °C, a single stirred tank reactor of 3.8 m^3^ ensures 60% KER conversion when 2500 kg/h KER are fed to the process (Table S9) with a solvent/feed mass ratio of 50:1.

### 3.5 Recovering and Recycling of the Solvent. Process Integration and Economic Analysis

**Figure 10:**
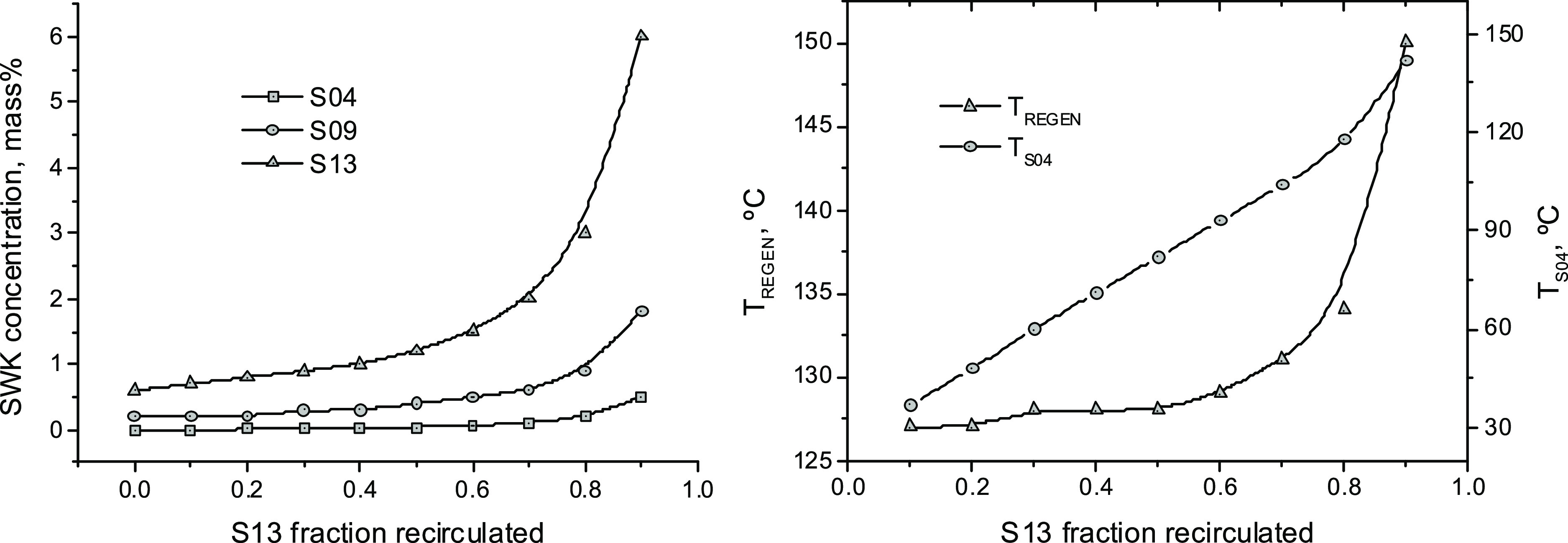
Mass fraction of the soluble products of the keratin decomposition (SWK) in different mixtures of the process to dissolve and regenerate keratin from chicken feathers with the (NaAc + urea) DES after removing the water contained in S09, for different fractions (w/w) of solvent recycled [left]. Temperatures at REGEN (S11) and S04 as a function of the solvent weight fraction recycled [right]. For the nomenclature, see Figure 2. The remaining process specifications correspond to the base case.

The water removal from the soluble mixture (S09) obtained after the keratin regeneration (Figures 1 and 2) and the subsequent recycling of the solvent recovered reduce both the demand of fresh solvent and the preheating necessities in the keratin dissolution section of the process. Thus, for the base case (Table 1), it was found that under recycling (S14) 80% of the solvent recovered (S13), *m*_S02_ ≈ 25.0 t/h and *T*_S04_ ≈ 116.5 °C (Tables S11–S13). However, recycling the solvent recovered causes the accumulation of the soluble, low-volatile products of the keratin decomposition (SWK) in the solvent. Their concentrations increase with the fraction (split fraction at the SPLITTER) of the solvent recycled (Figure 10).

Consequently, both the S04 temperature and the operating temperature at the REGEN increase with the fraction of the solvent recirculated (Figure 10). The mass fraction of the SWK in the recovered solvent and the REGEN operating temperature increase moderately up to approximately 70% S13 fraction recirculated but grow abruptly for higher split fractions at the SPLITTER (Figure 10).

For the conditions of the base case considered in this work (Table 1), about 415 t/h of a mixture (S09), containing 27.0 wt % of the DES, (0.2–1.8) wt % SWK, and (72.8–71.1) wt % water, depending on the fraction of the solvent recovered that has been recycled (Tables S11–S13), are fed to the solvent recovery section of the process (Figure 2).

The heat conditioning of these mixtures (SEP-HEAT) before the separation requires a high level of both energy consumptions, (approximately 205 MW thermal power, Table S14) and heat exchange surface areas (approximately 1985 m^2^; Tables S15 and S16). Furthermore, a high-volume separator (REGEN) of ca. 810 m^3^ is necessary to recover the DES solvent (Table S16) from its mixture with water. Thus, the DES recovery section is responsible simultaneously for 83.5% of the utilities and 44.2% of the equipment costs (Tables S14, S17 and S18, Figure S15) in the integrated process (Figure 2).

Interestingly, the utilities’ costs decrease monotonously with the increasing of the fraction of the solvent (SPLITT value) recycled to the process, while the equipment costs show a minimum value for SPLITT = 0.6 (Figure S13). However, variations of both the equipment and utilities’ costs with the fraction of the solvent recycled with respect to the base case (SPLITT = 0) are always lower than 5%.

The calculated unitary cost of the process, considering only the purchased cost of the equipment and the utilities’ costs, was $274/t feathers treated for SPLITT = 0.6 and the remainder conditions of the base case (Table 1).

From the two previous results, it could be concluded that the high solvent (S03/S01 mass ratio) and water (S08/S07 mass ratio) excesses used in the base case (Table 1) are responsible for the elevated total costs of the process.

### 3.6 Process Improvements and Cost Reductions

Reducing the excesses of both the solvent (S03/S01 mass ratio) used for dissolving the keratin and the water (S08/S07 mass ratio) for its regeneration (alternative cases, Table 1) significantly alters the mass and heat balances of the process (Tables S19–S22). The equipment sizing (Table S23) as well as the utilities’ and equipment costs (Tables S22 and S24) and, correspondingly, the total cost of the process (Tables S25 and S26) are diminished.

**Figure 11:**
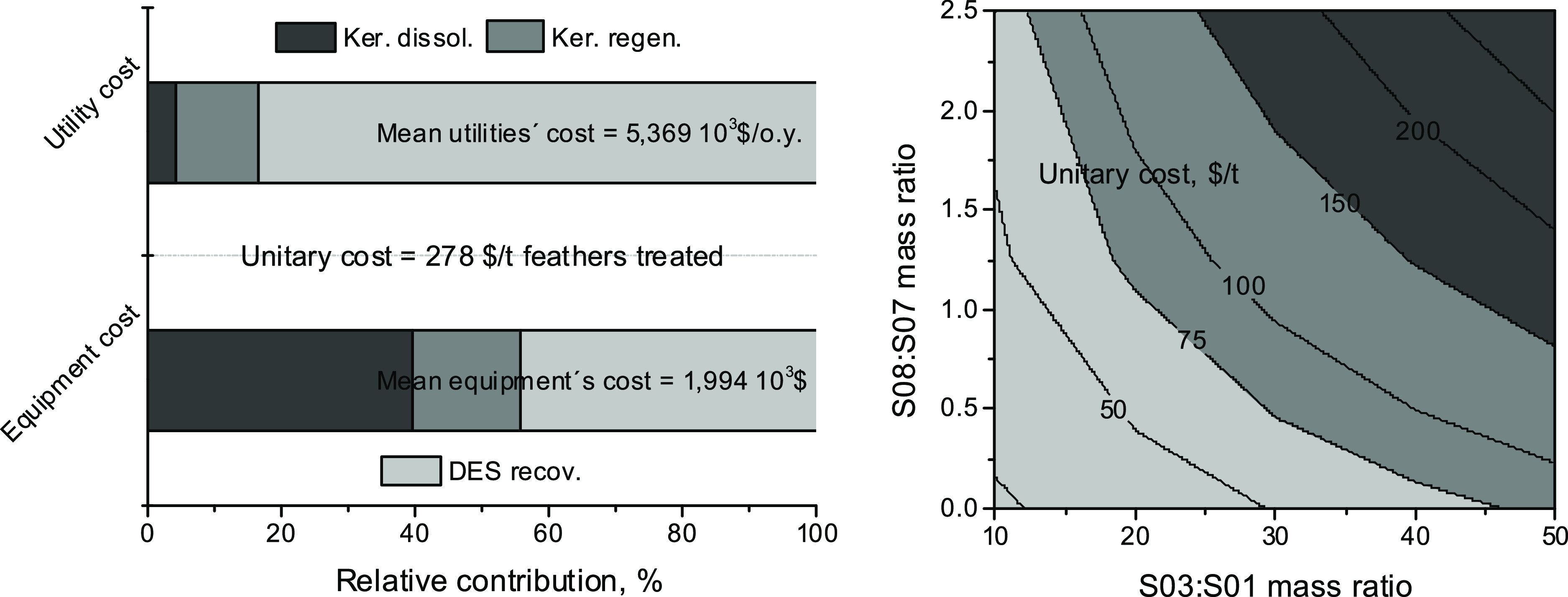
[Left, base case] Relative contributions of the different sections of the process to dissolve and regenerate keratin from chicken feathers using (NaAc + urea) as the reacting solvent on both the utility and purchased equipment costs. The current results are the mean costs for the interval 0–0.8 of DES recycled (SPLITT fraction). [Right, alternative cases] Unitary costs (total costs per ton of feathers treated) of the process with respect to both the solvent (S03/S01 mass ratio) and water (S08/S07 mass ratio) excesses used. Total costs include only the purchased cost of the equipment and the utility cost.

The unitary costs of the process are lowered up to ca. 20 $/t feathers treated (Tables S25 and S26; Figure 11), considering only the purchased equipment cost and the utilities’ cost. However, the diminishment of the S03/S01 mass ratio below 20:1 causes an undesirable increment of the REGEN operating temperature (Figure S14 and Tables S19–S21), which could produce the loss of a certain amount of the DES solvent by the vapor phase (Table S19).

Based on all the previous results, a final (nonoptimized, nonenergy integrated) process design was proposed (Figure S15), where a tubular reactor operating in adiabatic conditions (*T*_S05_ = 130 °C) and 4 bar pressure was selected for the keratin dissolution stage. To guarantee the operating pressure in the reactor, a pump was required. S03/S01 and S08/S07 mass ratios were set to 20:1 and 1:1, respectively, for avoiding the potential solvent losses (Table S27).

The unitary cost of the process dropped to approximately 58.5 $/t feathers treated (Tables S28–S31), which represents a significant decrease of 78.6% of the cost with respect to the base case (Table 1). For the economic scenario defined in the current work, the cost of the utilities represents, approximately, 95% of the total annual costs of the process. In this regard, it is important to remark that the total heating necessities of the process (estimated at approximately 75.8 MW) are much greater than the cooling necessities (estimated at approximately 5.7 MW) even considering the vapor generated at the REGEN process as a subproduct (as explained before) with a positive contribution to the economic balance of the process. This fact limits the real contribution of the heat integration to the process improvement and makes it highly heating-demanding. In fact, a possible heat exchanger intercrossing S06 and S09 streams (Figure 2) achieved an increase in the temperature of S11 only to 68 °C for the base case (Table S15). Moreover, the utilities’ costs are determined to be 81% (Figure S16) by the DES recovery section which is due to (i) the even large excesses of both the reacting solvent and the water used in the process and (ii) the strong interactions of this kind of solvent with the remainder components involved in the process.

## 4 Concluding Remarks

Structural models of keratin and the products of its decomposition, having molecular weights in the interval between approximately 1400 and 3000 g/mol, were created by sequencing conveniently the most abundant amino acids in the protein composition, followed by a geometry optimization using theoretical and quantum chemical methods. They reproduced qualitatively important features of the chemical process used to dissolve and regenerate the keratin from chicken feathers using the (NaAc + urea) DES mixture with water. A physically consistent model of the (NaAc + urea) DES was obtained via cluster configuration instead of considering it a simple mixture of the individual components.

The molecular properties derived from the previous structures were used to create a model of the dissolution and regeneration of keratin from chicken feathers in Aspen Plus by using the property model COSMOSAC implemented in the program. The process proposed included solvent recovery and recycling. The process model was completed with a pseudo-first-order kinetic equation obtained by processing conveniently the results of laboratory experiments already published. The kinetics of this process is characterized by a high energy activation of 104.9 kJ/mol.

The base case, specified in this work as closely as possible to the reaction conditions used in the laboratory experiments, entails unitary costs of approximately $280/t of feathers treated considering just the equipment and utilities’ costs. These elevated costs are mainly determined by the energy demand of the solvent recovery section of the process, which is determined by two factors: (i) the high excesses of both the solvent used to dissolve keratin and the water to regenerate keratin and (ii) the strong interaction of the reacting solvent with the remainder components of the process in the liquid phase. The solvent recovery section of the process is responsible for approximately 85% of the utilities’ costs and 45% of the equipment costs.

The total costs of the process can be reduced by ca. 78%, up to approximately 58.5 $/t feathers treated, diminishing the excesses of both the solvent and the water used in the process from 50:1 to 20:1 for the solvent and from 2.5:1 to 1:1 for the water (both in mass basis) and modifying the configuration of the keratin dissolution section using a tubular reactor operating in adiabatic regime with *T*_inlet_ = 130 °C and *P* = 4 bar instead of a stirred tank under isothermal conditions at atmospheric pressure. Nevertheless, the solvent excess cannot be reduced arbitrarily because it causes an increase of the operating temperature in the solvent regeneration column.

Although the experimental information available on the different operations of this process is not sufficient for creating and validating a more rigorous process model, the current results can be taken as sufficiently solid guidance to derive some important suggestions for its further development. First, it seems to be necessary to use the lowest possible solvent excesses in the process. For this, it is recommended to find new solvents able to interact more selectively with the disulfide bonds of keratin but simultaneously to interact as weakly as possible with water for reducing the energy consumed in the solvent recovery stage of the process. This seems to be a plausible alternative, taking into consideration that both the ILs and the DESs can be optimized for a specific application. However, the solvent design tasks should incorporate the corresponding process simulation, using models such as those employed in this work, as a solution to get an integrated techno-economic view of the process.

## Supporting Information Available

Additional data on the molecular modeling, property estimation, and process simulations (PDF)

## References

[ref1] Tesfaye T.; Sithole B.; Ramjugernath D.; Chunilall V. Valorisation of chicken feathers: Characterisation of chemical properties. Waste Manage. 2017, 68, 626–635. 10.1016/j.wasman.2017.06.050. 28687152

[ref2] Tesfaye T.; Sithole B.; Ramjugernath D.; Chunilall V. Valorisation of chicken feathers: Characterisation of physical properties and morphological structure. J. Cleaner Prod. 2017, 149, 349–365. 10.1016/j.jclepro.2017.02.112. 28687152

[ref3] Wang B.; Yang W.; McKittrick J.; Meyers M. A. Keratin: Structure, mechanical properties, occurrence in biological organisms, and efforts at bioinspiration. Prog. Mater. Sci. 2016, 76, 229–318. 10.1016/j.pmatsci.2015.06.001.

[ref4] Karthikeyan R.; Balaji S.; Sehgal P. K. Industrial applications of keratins. A review. J. Sci. Ind. Res. 2007, 66 (9), 710–715.

[ref5] Shavandi A.; Silva T. H.; Bekhit A. A.; Bekhit A. E.-D. A. Keratin: dissolution, extraction and biomedical application. Biomater. Sci. 2017, 5 (9), 1699–1735. 10.1039/C7BM00411G. 28686242

[ref6] Marzinek J. K.; Lian G.; Marzinek J. K.; Mantalaris A.; Pistikopoulos E. N.; Zhao Y.; Han L.; Chen L.; Bond P. J.; Noro M. G. Molecular and thermodynamic basis for EGCG-keratin interaction-Part I: Molecular dynamics simulations. AIChE J. 2013, 59 (12), 4816–4823. 10.1002/aic.14220.

[ref7] Arai K. M.; Takahashi R.; Yokote Y.; Akahane K. Amino-acid sequence of feather keratin from fowl. Eur. J. Biochem. 1983, 132 (3), 501–507. 10.1111/j.1432-1033.1983.tb07389.x. 6189711

[ref8] Calvaresi M.; Eckhart L.; Alibardi L. The molecular organization of the beta-sheet region in Corneous beta proteins (beta-keratins) of sauropsids explains its stability and polymerization into filaments. J. Struct. Biol. 2016, 194 (3), 282–291. 10.1016/j.jsb.2016.03.004. 26965557

[ref9] Bray D. J.; Walsh T. R.; Noro M. G.; Notman R. Complete structure of an epithelial keratin dimer: Implications for intermediate filament assembly. PLoS One 2015, 10 (7), e0132706–e0132722. 10.1371/journal.pone.0132706. 26181054 PMC4504709

[ref10] Alberts B.; Bray D.; Lewis J.; Raff M.; Roberts K.; Watson J. D. Molecular Biology of the Cell, 3rd ed.: Garland. New York, 1994.

[ref11] McKittrick J.; Chen P. Y.; Bodde S. G.; Yang W.; Novitskaya E. E.; Meyers M. A. The structure, functions, and mechanical properties of keratin. JOM 2012, 64 (4), 449–468. 10.1007/s11837-012-0302-8.

[ref12] Shavandi A.; Carne A.; Bekhit A. A.; Bekhit A. E.-D. A. An improved method for solubilisation of wool keratin using peracetic acid. J. Environ. Chem. Eng. 2017, 5 (2), 1977–1984. 10.1016/j.jece.2017.03.043.

[ref13] Earland C.; Knight C. S. Studies on the struture of keratin. 1. The analysis of fractions isolated from wool oxidized with peracetic acid. Biochim. Biophys. Acta 1955, 17 (4), 457–461. 10.1016/0006-3002(55)90406-6. 13249991

[ref14] Isarankura Na Ayutthaya S.; Tanpichai S.; Wootthikanokkhan J. Keratin extracted from chicken feather waste: Extraction, preparation, and structural characterization of the keratin and keratin/biopolymer films and electrospuns. J. Polym. Environ. 2015, 23 (4), 506–516. 10.1007/s10924-015-0725-8.

[ref15] Sinkiewicz I.; Sliwinska A.; Staroszczyk H.; Kolodziejska I. Alternative methods of preparation of soluble keratin from chicken feathers. Waste Biomass Valorization 2017, 8 (4), 1043–1048. 10.1007/s12649-016-9678-y.

[ref16] Khumalo M.; Sithole B.; Tesfaye T. Valorisation of waste chicken feathers: Optimisation of keratin extraction from waste chicken feathers by sodium bisulphite, sodium dodecyl sulphate and urea. J. Environ. Manag. 2020, 262 (110329), 110329–110337. 10.1016/j.jenvman.2020.110329. 32250808

[ref17] Nuutinen E.-M.; Willberg-Keyriläinen P.; Virtanen T.; Mija A.; Kuutti L.; Lantto R.; Jääskeläinen A. S. Green process to regenerate keratin from feathers with an aqueous deep eutectic solvent. RSC Adv. 2019, 9 (34), 19720–19728. 10.1039/C9RA03305J. 35519403 PMC9065387

[ref18] Zhang Z.; Nie Y.; Zhang Q.; Liu X.; Tu W.; Zhang X.; Zhang S. Quantitative change in disulfide bonds and microstructure variation of regenerated wool keratin from various ionic liquids. ACS Sustainable Chem. Eng. 2017, 5 (3), 2614–2622. 10.1021/acssuschemeng.6b02963.

[ref19] Idris A.; Vijayaraghavan R.; Patti A. F.; MacFarlane D. R. Distillable protic ionic liquids for keratin dissolution and recovery. ACS Sustainable Chem. Eng. 2014, 2 (7), 1888–1894. 10.1021/sc500229a.

[ref20] Idris A.; Vijayaraghavan R.; Rana U. A.; Fredericks D.; Patti A. F.; MacFarlane D. R. Dissolution of feather keratin in ionic liquids. Green Chem. 2013, 15 (2), 525–534. 10.1039/c2gc36556a.

[ref21] Ji Y.; Chen J.; Lv J.; Li Z.; Xing L.; Ding S. Extraction of keratin with ionic liquids from poultry feather. Sep. Purif. Technol. 2014, 132, 577–583. 10.1016/j.seppur.2014.05.049.

[ref22] Wang Y.-X.; Cao X.-J. Extracting keratin from chicken feathers by using a hydrophobic ionic liquid. Process Biochem. 2012, 47 (5), 896–899. 10.1016/j.procbio.2012.02.013.

[ref23] Li R.; Wang D. Preparation of regenerated wool keratin films from wool keratin-ionic liquid solutions. J. Appl. Polym. Sci. 2013, 127 (4), 2648–2653. 10.1002/app.37527.

[ref24] Sun P.; Liu Z. T.; Liu Z. W. Particles from bird feather: a novel application of an ionic liquid and waste resource. J. Hazard. Mater. 2009, 170 (2–3), 786–790. 10.1016/j.jhazmat.2009.05.034. 19497665

[ref25] Apostolidou C. Regenerated hoof keratin from 1-ethyl-3-methylimidazolium acetate and insights into disulfide-ionic liquid interactions from MD simulation. ChemistryOpen 2020, 9 (6), 695–702. 10.1002/open.202000096. 32528792 PMC7280737

[ref26] Ghosh A.; Clerens S.; Deb-Choudhury S.; Dyer J. M. Thermal effects of ionic liquid dissolution on the structures and properties of regenerated wool keratin. Polym. Degrad. Stab. 2014, 108, 108–115. 10.1016/j.polymdegradstab.2014.06.007.

[ref27] Freemantle M. Designer solvents - Ionic liquids may boost clean technology development. C&EN 1998, 76 (13), 32–37. 10.1021/cen-v076n013.p032.

[ref28] F Fernandez J.; Neumann J.; Thoming J. Regeneration, recovery and removal of ionic liquids. Curr. Org. Chem. 2011, 15 (12), 1992–2014. 10.2174/138527211795703676.

[ref29] Dai Y.; van Spronsen J.; Witkamp G.-J.; Verpoorte R.; Choi Y. H. Ionic liquids and deep eutectic solvents in natural products research: mixtures of solids as extraction solvents. J. Nat. Prod. 2013, 76 (11), 2162–2173. 10.1021/np400051w. 24188074

[ref30] Smith E. L.; Abbott A. P.; Ryder K. S. Deep eutectic solvents (DESs) and their applications. Chem. Rev. 2014, 114 (21), 11060–11082. 10.1021/cr300162p. 25300631

[ref31] Wahlstrom R.; Rommi K.; Willberg-Keyrilainen P.; Ercili-Cura D.; Holopainen-Mantila U.; Hiltunen J.; Makinen O.; Nygren H.; Mikkelson A.; Kuutti L. High yield protein extraction from brewer’s spent grain with novel carboxylate salt-urea aqueous deep eutectic solvents. ChemistrySelect 2017, 2 (29), 9355–9363. 10.1002/slct.201701492.

[ref32] Gutierrez A.; Atilhan M.; Aparicio S. Theoretical study of oil desulfuration by ammonium-based deep eutectic solvents. Energy Fuels 2018, 32 (7), 7497–7507. 10.1021/acs.energyfuels.8b01403.

[ref33] Adeyemi I.; Sulaiman R.; Almazroui M.; Al-Hammadi A.; AlNashef I. M. Removal of chlorophenols from aqueous media with hydrophobic deep eutectic solvents: Experimental study and COSMO RS evaluation. J. Mol. Liq. 2020, 311 (113180), 113180–113211. 10.1016/j.molliq.2020.113180.

[ref34] Pan Y.; Alam M. A.; Wang Z.; Huang D.; Hu K.; Chen H.; Yuan Z. One-step production of biodiesel from wet and unbroken microalgae biomass using deep eutectic solvent. Bioresour. Technol. 2017, 238, 157–163. 10.1016/j.biortech.2017.04.038. 28433903

[ref35] Procentese A.; Raganati F.; Olivieri G.; Russo M. E.; Rehmann L.; Marzocchella A. Low-energy biomass pretreatment with deep eutectic solvents for bio-butanol production. Bioresour. Technol. 2017, 243, 464–473. 10.1016/j.biortech.2017.06.143. 28688330

[ref36] Xu H.; Kong Y.; Peng J.; Song X.; Liu Y.; Su Z.; Li B.; Gao C.; Tian W. Comprehensive analysis of important parameters of choline chloride-based deep eutectic solvent pretreatment of lignocellulosic biomass. Bioresour. Technol. 2021, 319 (124209), 124209–124213. 10.1016/j.biortech.2020.124209. 33045547

[ref37] Xin K.; Hashish M.; Roghair I.; van Sint Annaland M. Process simulation and economic analysis of pre-combustion CO_2_ capture with deep eutectic solvents. Front. Energy Res. 2020, 8, 1–13. 10.3389/fenrg.2020.573267.

[ref38] Zhang W.; Ma Z.; Liu X.; Liu Y.; Hou Z.; Qi J.; Ma Y.; Wang L.; Wang Y. Molecular mechanism and absorption performance evaluation of CO_2_ capture from the PCC process by monoethanolamine-based deep eutectic solvents. Ind. Eng. Chem. Res. 2021, 60 (3), 1483–1493. 10.1021/acs.iecr.0c04970.

[ref39] Haider J.; Qyyum M. A.; Kazmi B.; Ali I.; Nizami A.-S.; Lee M. Simulation study of deep eutectic solvent-based biogas upgrading process integrated with single mixed refrigerant biomethane liquefaction. Biofuel Res. J. 2020, 7 (4), 1245–1255. 10.18331/BRJ2020.7.4.3.

[ref40] Ferro V. R.; Ruiz E.; de Riva J.; Palomar J. Introducing process simulation in ionic liquids design/selection for separation processes based on operational and economic criteria through the example of their regeneration. Sep. Purif. Technol. 2012, 97, 195–204. 10.1016/j.seppur.2012.02.026.

[ref41] Garcia-Chavez L. Y.; Schuur B.; de Haan A. B. Conceptual process design and economic analysis of a process based on liquid-liquid extraction for the recovery of glycols from aqueous streams. Ind. Eng. Chem. Res. 2013, 52 (13), 4902–4910. 10.1021/ie303187x.

[ref42] Wu Z.; Liu C.; Cheng H.; Chen L.; Qi Z. Tuned extraction and regeneration process for separation of hydrophobic compounds by aqueous ionic liquid. J. Mol. Liq. 2020, 308 (113032), 113032–113039. 10.1016/j.molliq.2020.113032.

[ref43] Ferro V. R.; Ruiz E.; Tobajas M.; Palomar J. F. Integration of COSMO-based methodologies into commercial process simulators: Separation and purification of reuterin. AIChE J. 2012, 58 (11), 3404–3415. 10.1002/aic.13746.

[ref44] Klamt A. The COSMO and COSMO-RS solvation models. Wiley Interdiscip. Rev.: Comput. Mol. Sci. 2018, 8 (1), e1338 10.1002/wcms.1338.

[ref45] Naik P. K.; Paul S.; Banerjee T. Liquid liquid equilibria measurements for the extraction of poly aromatic nitrogen hydrocarbons with a low cost deep eutectic solvent: Experimental and theoretical insights. J. Mol. Liq. 2017, 243, 542–552. 10.1016/j.molliq.2017.08.044.

[ref46] Verma R.; Banerjee T. Liquid-liquid extraction of lower alcohols using menthol-based hydrophobic deep eutectic solvent: Experiments and COSMO-SAC predictions. Ind. Eng. Chem. Res. 2018, 57 (9), 3371–3381. 10.1021/acs.iecr.7b05270.

[ref47] Benguerba Y.; Alnashef I. M.; Erto A.; Balsamo M.; Ernst B. A quantitative prediction of the viscosity of amine based DESs using S sigma-profile molecular descriptors. J. Mol. Struct. 2019, 1184, 357–363. 10.1016/j.molstruc.2019.02.052.

[ref48] Aldawsari J. N.; Adeyemi I. A.; Bessadok-Jemai A.; Ali E.; AlNashef I. M.; Hadj-Kali M. K. Polyethylene glycol-based deep eutectic solvents as a novel agent for natural gas sweetening. PLoS One 2020, 15 (9), e0239493 10.1371/journal.pone.0239493. 32956424 PMC7505472

[ref49] Paduszynski K. An overview of the performance of the COSMO-RS approach in predicting the activity coefficients of molecular solutes in ionic liquids and derived properties at infinite dilution. Phys. Chem. Chem. Phys. 2017, 19 (19), 11835–11850. 10.1039/C7CP00226B. 28435940

[ref50] Diedenhofen M.; Klamt A. COSMO-RS as a tool for property prediction of IL mixtures. A review. Fluid Phase Equilib. 2010, 294 (1–2), 31–38. 10.1016/j.fluid.2010.02.002.

[ref51] Ijardar S. P. Deep eutectic solvents composed of tetrabutylammonium bromide and PEG: Density, speed of sound and viscosity as a function of temperature. J. Chem. Thermodyn. 2020, 140, 105897 10.1016/j.jct.2019.105897.

[ref52] Ferreira E. S. C.; Voroshylova I. V.; Figueiredo N. M.; Pereira C. M.; Cordeiro M. N. D. S. Computational and experimental study of propeline: A choline chloride based deep eutectic solvent. J. Mol. Liq. 2020, 298 (111978), 111978–112011. 10.1016/j.molliq.2019.111978.

[ref53] Baz J.; Held C.; Pleiss J.; Hansen N. Thermophysical properties of glyceline- water mixtures investigated by molecular modelling. Phys. Chem. Chem. Phys. 2019, 21 (12), 6467–6476. 10.1039/C9CP00036D. 30840001

[ref54] Ferro V. R.; Moya C.; Moreno D.; Santiago R.; de Riva J.; Pedrosa G.; Larriba M.; Diaz I.; Palomar J. Enterprise ionic liquids database (ILUAM) for use in Aspen ONE programs suite with COSMO-based property methods. Ind. Eng. Chem. Res. 2018, 57, 980–989. 10.1021/acs.iecr.7b04031.

[ref55] Paulechka Y. U.; Kabo A. G.; Blokhin A. V.; Kabo G. J.; Shevelyova M. P. Heat capacity of ionic liquids: Experimental determination and correlations with molar volume. J. Chem. Eng. Data 2010, 55 (8), 2719–2724. 10.1021/je900974u.

[ref56] Picard K.; Thomas D.; Festa-Bianchet M.; Belleville F.; Laneville A. Differences in thermal conductivity of tropical and temperate bovid horns. Ecoscience 1999, 6, 148–158. 10.1080/11956860.1999.11682515.

[ref57] Lin S. T.; Sandler S. I. A priori phase equilibrium prediction from a segment contribution solvation model. Ind. Eng. Chem. Res. 2002, 41 (5), 899–913. 10.1021/ie001047w.

[ref58] Meindersma W. G. W.; Onink F. S. A. F.; Hansmeier A. R.; de Haan A. B. Long term pilot plant experience on aromatics extraction with ionic liquids. Sep. Sci. Technol. 2012, 47 (2), 337–345. 10.1080/01496395.2011.620590.

[ref59] Jongmans M. T. G.; Trampé J.; Schuur B.; de Haan A. B. Solute recovery from ionic liquids: A conceptual design study for recovery of styrene monomer from [4-mebupy] [BF_4_]. Chem. Eng. Process. 2013, 70, 148–161. 10.1016/j.cep.2013.04.007.

[ref60] de Riva J.; Ferro V. R.; Moreno D.; Diaz I.; Palomar J. Aspen Plus supported conceptual design of the aromatic-aliphatic separation from low aromatic content naphtha using 4-methyl-N-butylpyridinium tetrafluoroborate ionic liquid. Fuel Process. Technol. 2016, 146, 29–38. 10.1016/j.fuproc.2016.02.001.

[ref61] Makos P.; Slupek E.; Gebicki J. Extractive detoxification of feedstocks for the production of biofuels using new hydrophobic deep eutectic solvents. Experimental and theoretical studies. J. Mol. Liq. 2020, 308 (113101), 113101–113111. 10.1016/j.molliq.2020.113101.

[ref62] Wang B.; Cheng J.; Wang D.; Li X.; Meng Q.; Zhang Z.; An J.; Liu X.; Li M. Study on the desulfurization and regeneration performance of functional deep eutectic solvents. ACS Omega 2020, 5 (25), 15353–15361. 10.1021/acsomega.0c01467. 32637809 PMC7331076

[ref63] Warrag S. E. E.; Darwish A. S.; Abuhatab F. O. S.; Adeyemi I. A.; Kroon M. C.; AlNashef I. M. Combined extractive dearomatization, desulfurization, and denitrogenation of oil fuels using deep eutectic solvents: A parametric study. Ind. Eng. Chem. Res. 2020, 59 (25), 11723–11733. 10.1021/acs.iecr.0c01360.

[ref64] Xue B.; Yang Y.; Tang R.; Xue D.; Sun Y.; Li X. Efficient dissolution of lignin in novel ternary deep eutectic solvents and its application in polyurethane. Int. J. Biol. Macromol. 2020, 164, 480–488. 10.1016/j.ijbiomac.2020.07.153. 32687900

[ref65] Ulrich G. D.; Vasudevan P. T. Chemical Engineering process design and economics. A Practical Guide, 2nd ed.; Process Publishing: Durham, NH, 2004.

[ref66] Busato M.; Migliorati V.; Del Giudice A.; Di Lisio V.; Tomai P.; Gentili A.; D’Angelo P. Anatomy of a deep eutectic solvent: structural properties of choline chloride: sesamol 1:3 compared to reline. Phys. Chem. Chem. Phys. 2021, 23 (20), 11746–11754. 10.1039/D1CP01105G. 33982713

[ref67] Tomasi J.; Persico M. Molecular Interactions in Solution: An Overview of Methods Based on Continuous Distributions of the Solvent. Chem. Rev. 1994, 94 (7), 2027–2094. 10.1021/cr00031a013.

[ref68] Tanner E. E. L.; Piston K. M.; Ma H.; Ibsen K. N.; Nangia S.; Mitragotri S. The influence of water on choline-based ionic liquids. ACS Biomater. Sci. Eng. 2019, 5 (7), 3645–3653. 10.1021/acsbiomaterials.9b00243. 33405745

[ref69] Ma C.; Laaksonen A.; Liu C.; Lu X.; Ji X. The peculiar effect of water on ionic liquids and deep eutectic solvents. Chem. Soc. Rev. 2018, 47 (23), 8685–8720. 10.1039/C8CS00325D. 30298877

[ref70] Liu X.; Nie Y.; Liu Y.; Zhang S.; Skov A. L. Screening of ionic liquids for keratin dissolution by means of COSMO-RS and experimental verification. ACS Sustainable Chem. Eng. 2018, 6 (12), 17314–17322. 10.1021/acssuschemeng.8b04830.

