# Supplementary material for: Multiscale Conceptual Design of a Scalable and Sustainable Process to Dissolve and Regenerate Keratin from Chicken Feathers
Source: Ind Eng Chem Res. 2023 Aug 16;62(34):13324–39. doi: 10.1021/acs.iecr.3c01843 (PMC10863073; doi:10.1021/acs.iecr.3c01843)
Supplement: Supplementary file 1 — ie3c01843_si_001.pdf [file ie3c01843_si_001.pdf]

Supporting Information

Multiscale conceptual design of a scalable and sustainable process to dissolve and regenerate the keratin from the chicken feathers.

Víctor R. Ferro[1*], Héctor Leiva[1], Erasmo Cadena[2], José Luis Valverde

1. Department of Chemical Engineering. Universidad Autónoma de Madrid. 28049 Madrid, Spain.

2. Department of Green Chemistry and Technology, Ghent University, 9000 Gent, Belgium.

3. Department of Chemical Engineering. Universidad de Castilla la Mancha, 13071 Ciudad Real, Spain.

* Corresponding author. E-mail: victor.ferro@uam.es. Tel. +34 91 497 7607

Experiment S1.

To evaluate the contribution of the H-bond interaction to the cluster stability, an alternative structure to the shown in Figure 3 where the H···O bonds were broken was also calculated. To do that, the urea molecules were disposed perpendicularly to the plane of the NaAc. The interatomic distances Na···O are practically identical to those obtained for the cluster of minimum energy (Figure 3 in the paper).

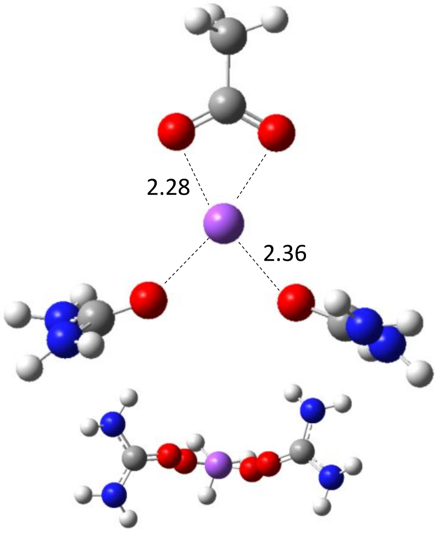

However, this second structure is significantly less stable than the first one. This energy difference could be related to the H-bond interactions that predominate in the *real* aggregate.

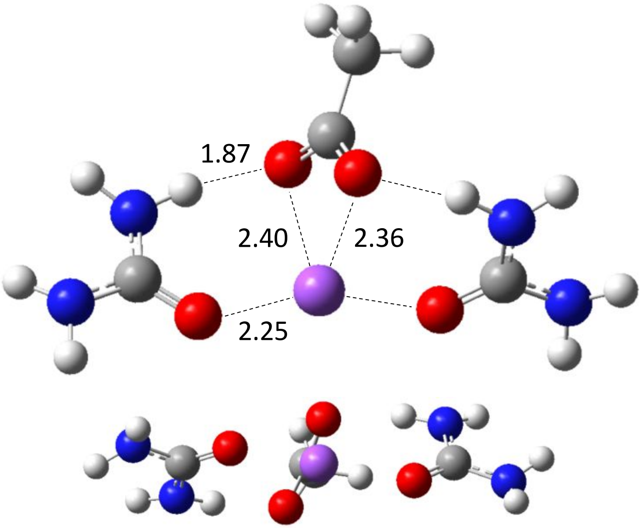

Figure S1. Solvent-phase optimized structure of the NaAc:Urea (1:2 molar ratio) DES considering interaction with water (ε = 80). SCRF calculation at m06-2x/6-311G(d,p) computational level in Gaussian 16.0. Superior view [Up]. Lateral view [Down].

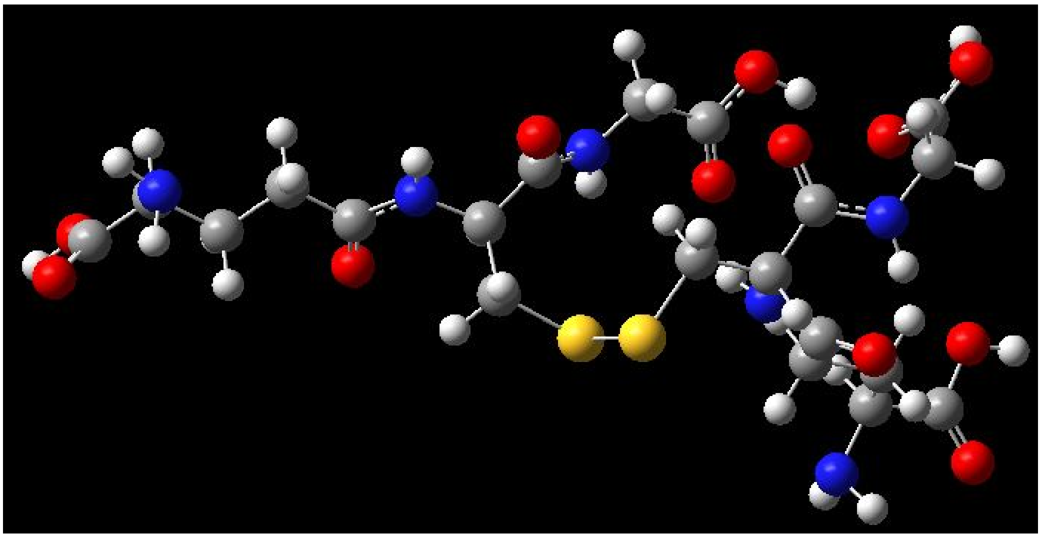

Figure S2. Optimized (m062x/6-311G(d,p) calculation level in Gaussian 16.0) structure of the oxidized glutathione (GSSG) obtained in the current work.

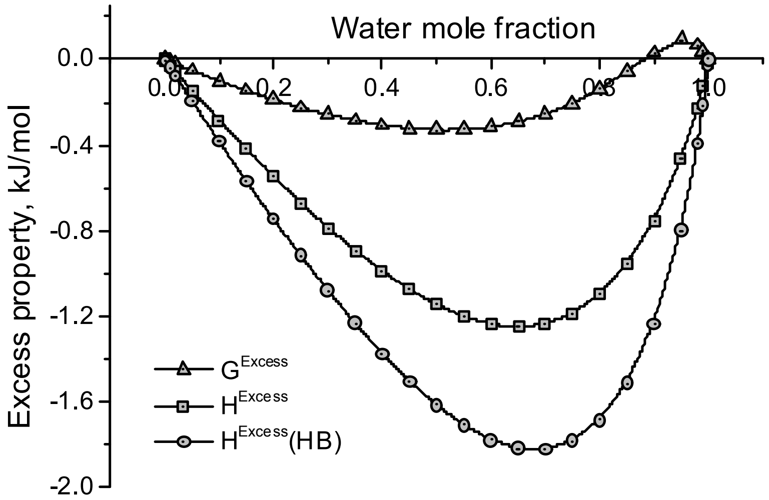

Figure S3. Excess free energy (G^{Excess}) and excess enthalpy (H^{Excess}) as function of the composition for mixtures (GSSG + water). Contribution of the H-bond interactions to H^{Excess} ($H^{Excess}(HB)$) COSMO-RS calculations. T = 298 K.

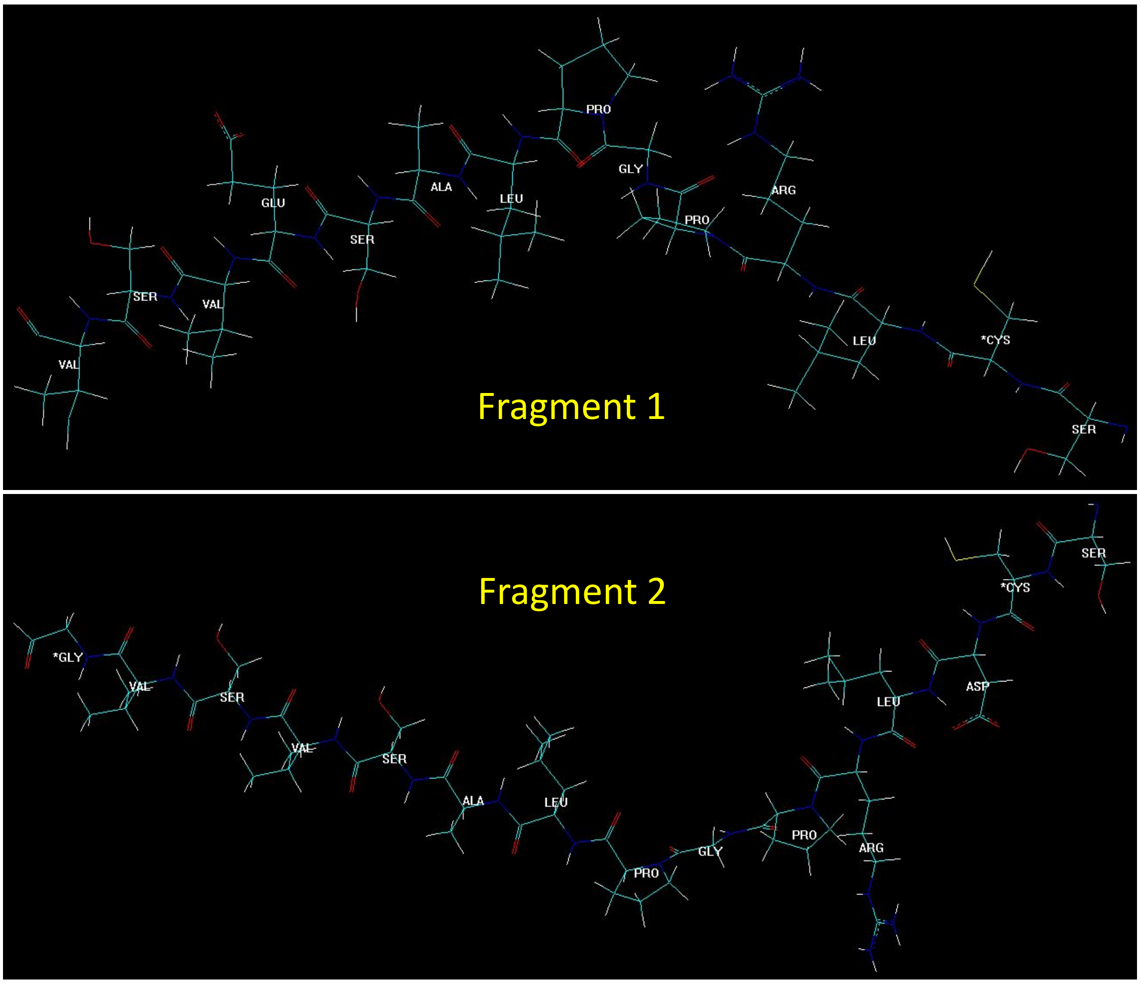

Figure S4. Input structures used in this work to create molecular models of the fragments resulting from the breaking of the keratin supramolecular structure.

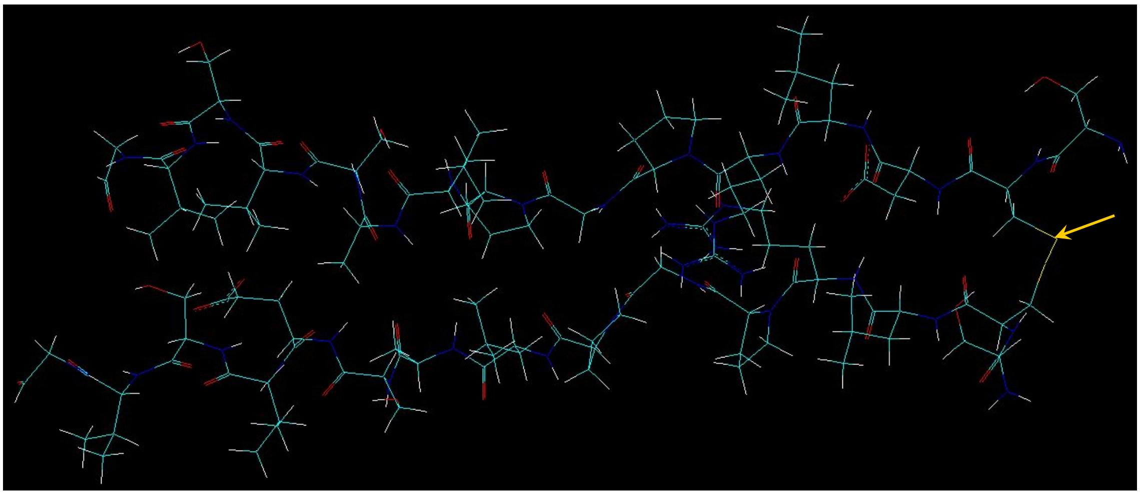

Figure S5. Input structure used in this work to create a molecular model of the keratin. A unique sulfur bond was included in the structure. It is shown by an arrow in the Figure.

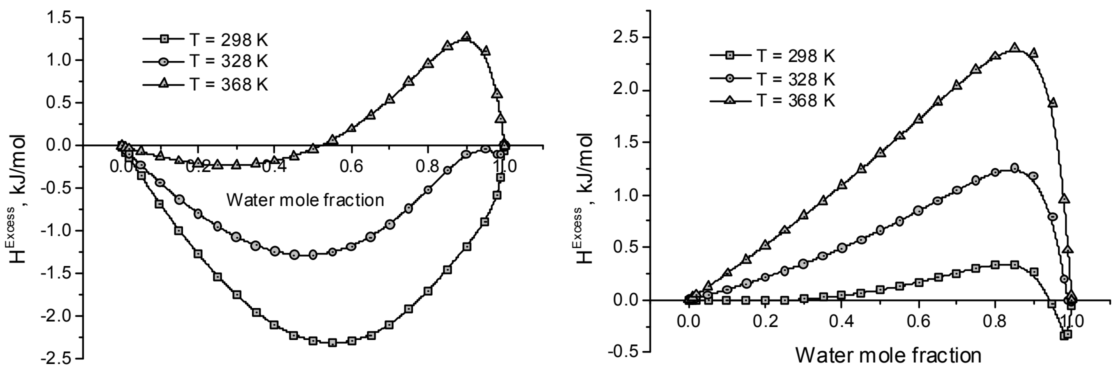

Figure S6. Excess enthalpies of mixtures (Keratin fragment + water) at different temperatures. Fragment (1) [Left]. Fragment (2) [Right]. COSMO-RS calculations. T = 298 K.

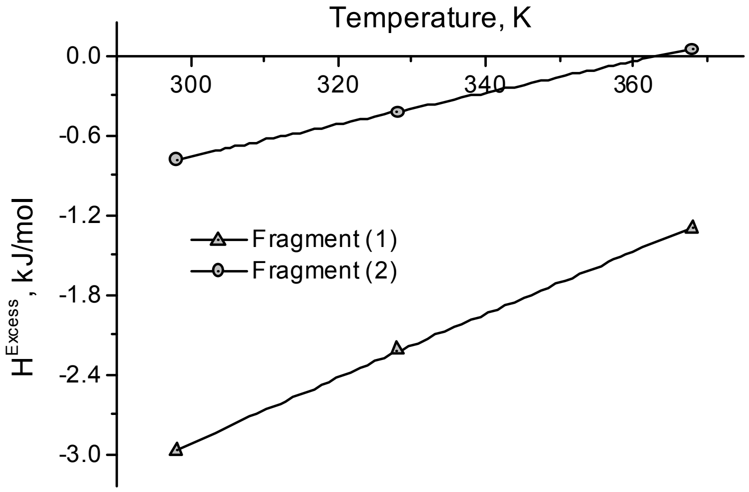

Figure S7. Influence of the temperature on the H-bond contribution to the excess enthalpy ($H^{Excess}(HB)$) in equimolar mixtures (Keratin fragment + water) for fragments (1) and (2). COSMO-RS calculations. T = 298 K.

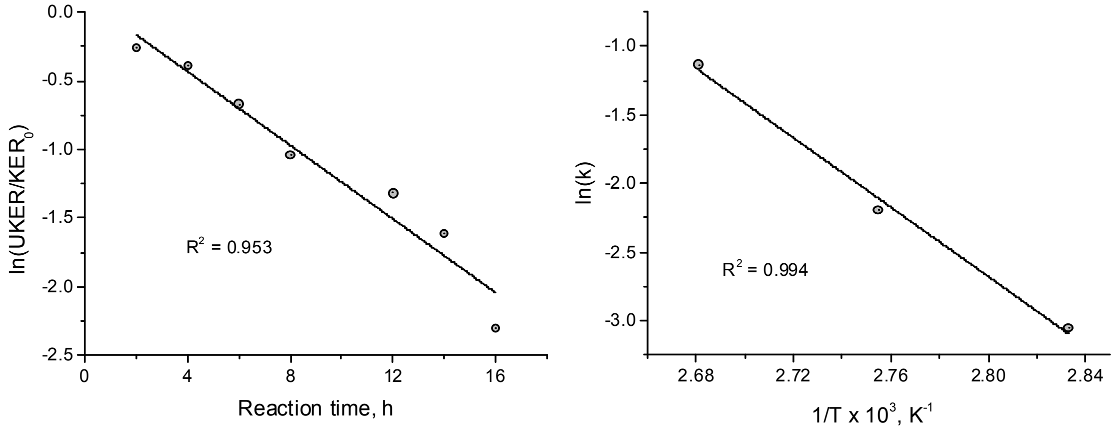

Figure S8. $ln\left(\frac{UKER}{KER_0}\right)$ vs. t [Left], ln(k) vs. 1/T [Right] in the experiments to dissolve the keratin from the chicken feathers with the (NaAc + Urea) DES. The raw kinetic data was taken from (Nuutinen et al., 2019). *UKER* and KER_0 mean, respectively, undissolved feathers and feathers fed to the process. k (h^{-1}) is the specific velocity of the process.

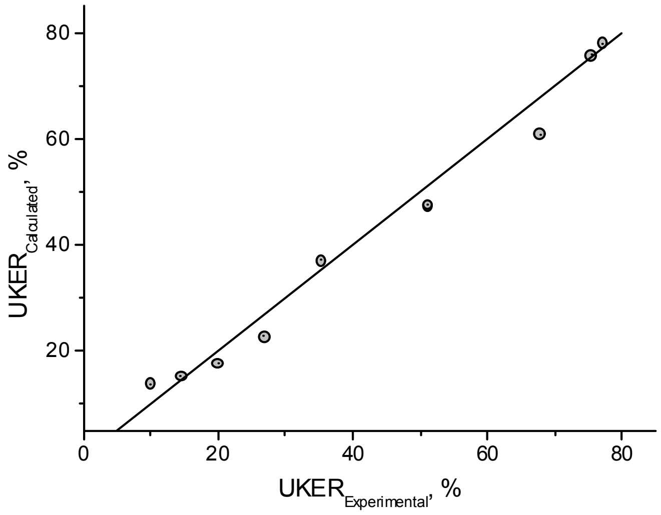

Figure S9. Comparison of the experimental and calculated fractions of undissolved feathers (*UKER*) for the feather mass, reaction times and temperatures used in laboratory experiments[1]. Calculations were carried out using the batch reactor model in Aspen Plus and the kinetic equation obtained in this work.

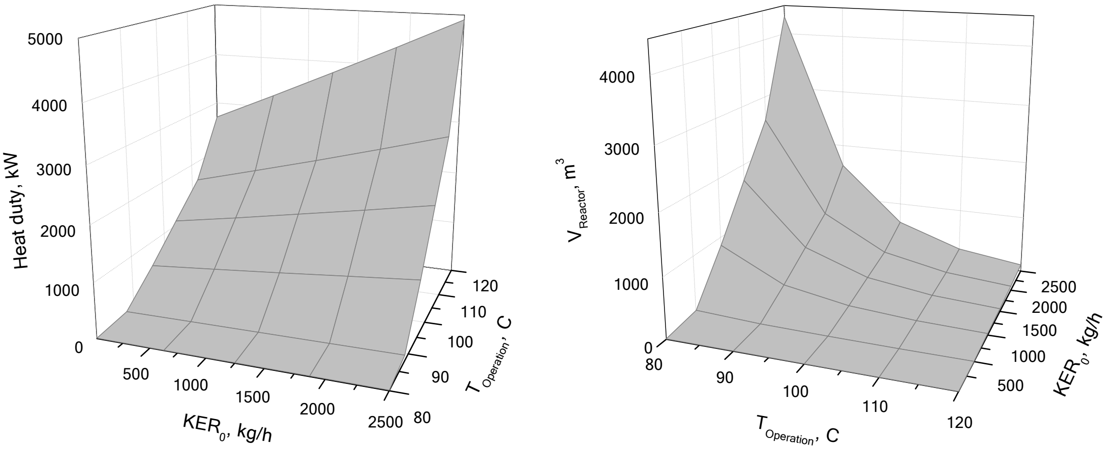

Figure S10. Dependencies of the heat necessities for thermal conditioning of raw materials up to reacting temperatures [Left] and reactor size [Right] respect to feather mass fed to the reactor and its operating temperatures. Solvent added to the reactor was considered the fresh (DES + water) mixture at 25 °C. The remainder specifications correspond to the Base Case (Table 1). Only the keratin dissolution section of the process proposed here (Figure 2) was considered.

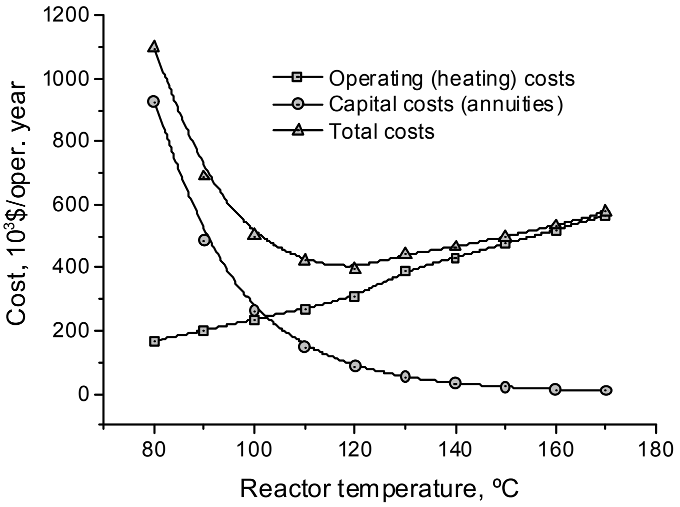

Figure S11. Capital, operative and total annual costs of the keratin dissolution in the (NaAc + Urea) DES mixture. The costs correspond exclusively to the keratin dissolution section of the process shown in Figure 2. Operating costs refer to the steam consumed in PRE-HEAT. Capital costs are related to the purchasing costs of the PRE-HEAT and REACTOR. Individual values are in Tables 8SM and 9SM. The solvent added was considered the fresh (DES + water) mixture at 25 °C. The remainder specifications correspond to the Base Case (Table 1).

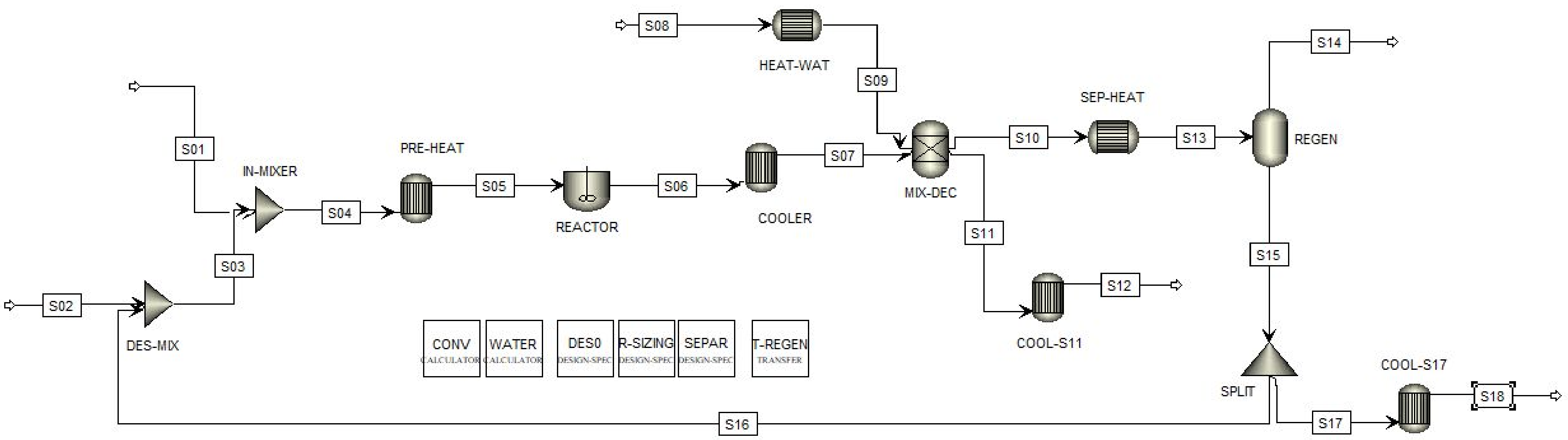

Figure S12. Aspen Plus model of the process devoted to dissolve and regenerate the keratin from chicken feathers using the (NaAc + Urea) DES as reacting solvent. The current flowsheet includes the issues omitted for simplicity in the Figure 2. Consequently, the heating/cooling utilities have been associated to the corresponding heat exchanger models. The streams numbering has been altered respect to Figure 2. The utilities specifications have been described in the paragraph Equipment sizing and cost estimation.

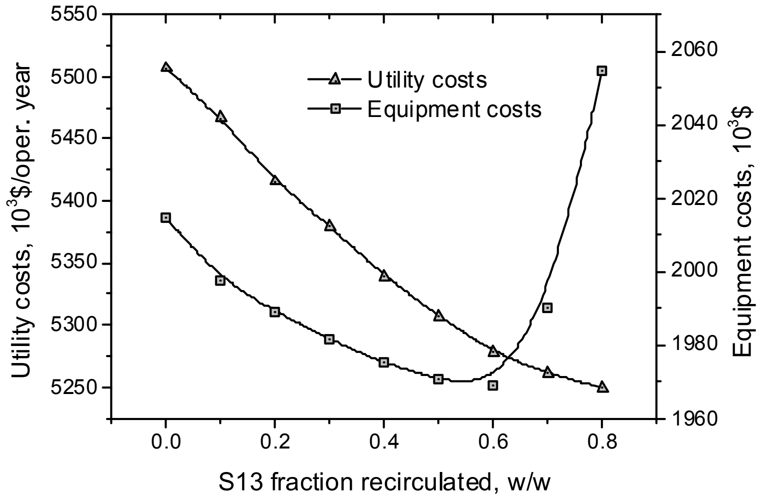

Figure S13. Utilities´ and purchased equipment´s costs as a function of the solvent recycled in the process to dissolve and regenerate the keratin from chicken feathers using the (NaAc + Urea) DES as reacting solvent. The remainder specifications correspond to the Base Case (Table 1). The stream identification matches with Figure 2; however, the cost estimation was supported by the process model shown in Figure S12.

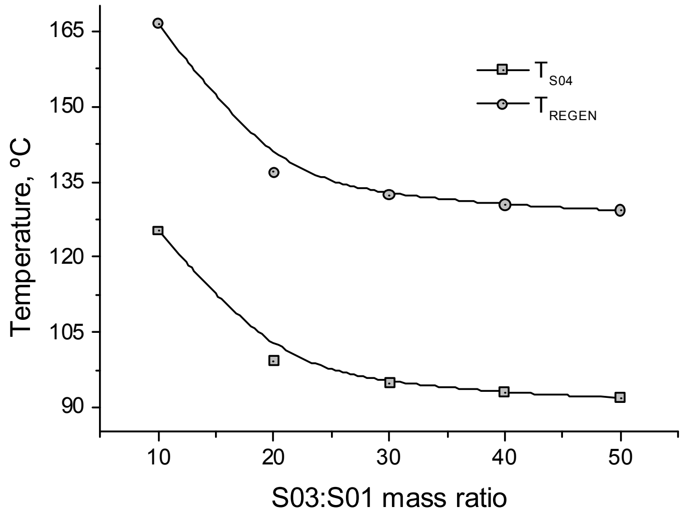

Figure S14. Temperature of stream S04 and operating temperature at the REGEN (Figure 2) in the process to dissolve and regenerate the keratin from the chicken feathers using the (NaAc + Urea) DES as reacting solvent respect to the solvent excess (S03:S01 mass ratio) used. The reminder specifications correspond to the Base Case (Table 1). The excess of water employed in the keratin regeneration does not affect these temperatures.

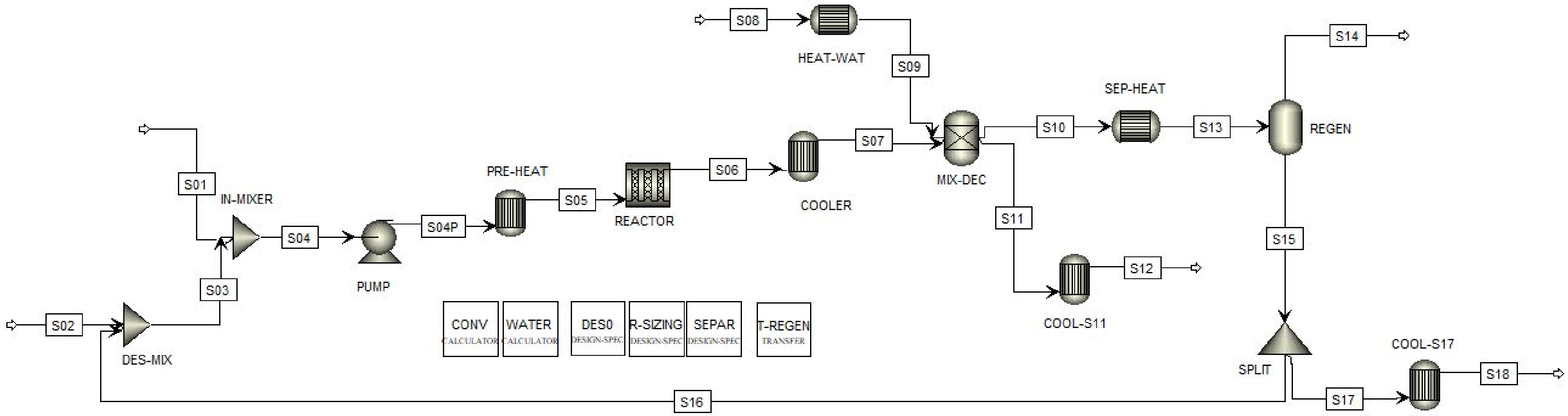

Figure S15. Flowsheet of the final process design proposed in this work to dissolve and regenerate the keratin from chicken feathers using the (NaAc + Urea) DES as reacting solvent. A tube reactor operating in adiabatic conditions was selected to model the keratin dissolution. A pump is needed because the reactor operates at pressures above the atmospheric pressure (4 bar). The current process diagram modifies the one shown in Figure S12.

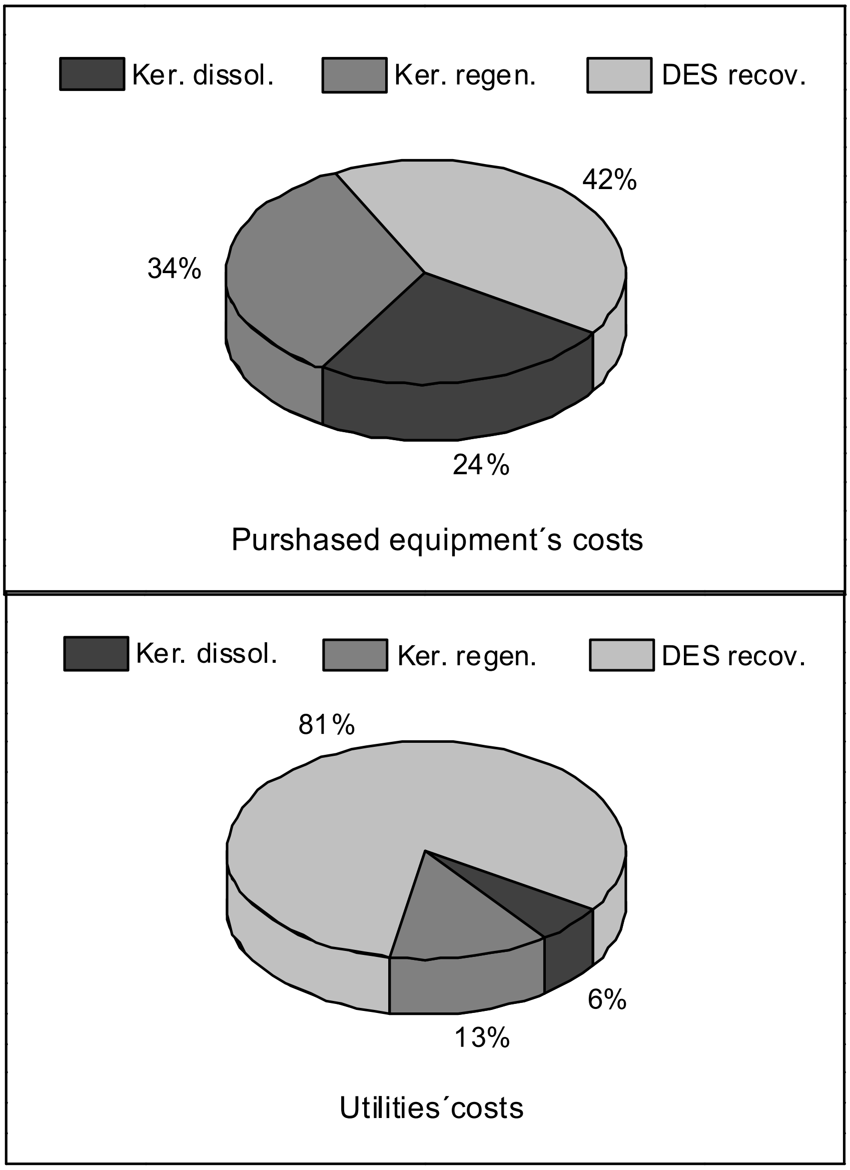

Figure S16: Contribution (in percentage) of each process section to the equipment´s [Up] and utilities´ [Down] costs in the final design of the process to dissolve and regenerate the keratin from the chicken feathers using the (NaAc + Urea) DES as reacting solvent. Calculations were supported by the process model shown in Figure S15.

Table S1. Experimental conditions and results of several laboratory experiments for dissolving and regenerating keratin from different keratinous materials using ionic liquids and deep eutectic solvents.

Reference	Keratinous raw material and solvent	Operating conditions	Process efficiency
1	Chicken feathers<br>10 wt% water + 90 wt% DES (NaAc + urea) molar ratio 1:2	3 g feathers + 147 g solvent<br>Temperature: 80 - 100 °C<br>Dissolution time: 2 - 24 h<br>350 mL water for keratin regeneration	Undissolved feathers 10 - 77 wt%<br>Up to ~ 70% disulfide bond cleavage
2	Goat wool<br>Several ionic liquids (ILs)	Wool:IL in proportion 1:10 (w/w)<br>Temperature: 80 - 100 °C<br>Dissolution time: 30 - 180 min.<br>Ethanol excess to regenerate the keratin	Regenerated keratin recovery 10 - 70 wt%<br>Up to ~ 90% disulfide bond cleavage
3	Turkey feathers<br>Protic ionic liquid, [DMEA][HCOO][(1)]	Keratin/IL solubility = 150 mg/g<br>Temperature: 100 °C<br>Dissolution time: 7 h	Regenerated keratin recovery up to 63 wt%
4	Duck feathers<br>Several ionic liquids<br>Na_2SO_3 was also added	Temperature: 80 - 90 °C<br>20 wt% water + IL solution 10 wt% Na_2SO_3<br>Solvent/feathers = 10 - 40:1 (wt/wt)<br>Dissolution time: 40 - 60 min.<br>Keratin regeneration with water	Dissolution of keratin up to 96%<br>Yield to regenerated keratin up to 70%
5	Fabric based on New Zealand Merino wool<br>[bmim][Cl][(2)] ionic liquid	Temperature: 120 - 180 °C<br>Solvent/feathers = 6:1 (wt/wt)<br>Dissolution time: 30 min.	Yields of regenerated keratin were 57% - 18% for temperatures in the interval 120 °C - 180 °C
6	Turkey feathers<br>Several aprotic ionic liquids	Temperature: 130 °C<br>Dissolution time: up to 10 h	Keratin solubility in ILs up to 45 wt%<br>Regenerated keratin recovery up to 51 - 59 wt%
7	Wool keratin<br>[bmim][Cl][(2)] and [amim][Cl][(3)] ionic liquids	Temperature: 130 °C<br>Dissolution time: up to 10 h	Feathers solubilities in IL up to 20 wt%[(5)]
8	Chicken feathers<br>Hydrophobic ionic liquid [HOemim][NTf_2][(4)]<br>$NaHSO_3$ was also added	Temperature: 70 - 100 °C<br>Solvent/feathers = 20:1 to 50:1 (wt/wt)<br>Dissolution time: 3 - 6 h<br>50 wt% water (respect to IL) for regeneration	Extraction yield of keratin (regenerated keratin) lower than 30% was obtained for all the conditions explored, approx. 14.5% for S:F[(6)] = 20:1 and 22.2% for S:F =40:1

[(1)] dimethylammonium formate. [(2)] 1-butyl-3-methylimidazolium chloride. [(3)] 1-allyl-3-methylimidazolium chloride. [(4)] 1-(2-hydroxyethyl)-3-methylimidazolium bis(trifluorometanesulfonyl)imide. [(5)] keratin regeneration from the solution with ionic liquids was not performed. [(6)] Some authors use this nomenclature as if it were a conventional liquid-liquid extraction.

Table S2. Amino acid composition (%mole[(1)]) of bird feathers.

Amino acid	Symbol	Ref. 1	Ref. 2	Ref. 3	Ref. 4[(2)]	Ref. 5	Ref. 6	Ref. 7	Ref. 8	Ref. 9[(3)]	Average	Std. Dev.
Serine	Ser	12.8	16.7	14.1	8.6	10.8	15.7	14.6	16.0	13.1	13.6	2.6
Glycine	Gly	13.8	11.5	13.7	14.0	6.0	11.5	10.8	11.0	11.5	11.5	2.4
Proline	Pro	12.6	12.5	9.8	11.4	5.3	11.7	10.7	12.0	9.4	10.6	2.3
Valine	Val	7.5	9.4	7.8	8.7	8.6	7.7	7.5	9.0	7.0	8.1	0.8
Leucine	Leu	6.8	6.3	8.3	9.2	9.7	7.4	7.7	6.0	6.9	7.6	1.3
Glutamic acid	Glu	8.3	2.1	6.9	6.8	9.2	8.6	8.5	7.0	6.8	7.1	2.1
Cysteine	Cys	6.1	7.3	7.8	8.5	6.5	4.2	8.8	7.0	7.3	7.1	1.4
Alanine	Ala	5.4	4.2	8.7	6.4	5.4	5.6	5.1	4.0	6.6	5.7	1.4
Aspartic acid	Asp	5.9	2.1	5.6	6.2	5.8	6.3	5.6	5.0	5.3	5.3	1.3
Glutamine	Gln	ND[(4)]	5.2	ND	ND	ND	ND	ND	ND	ND	5.2	1.7
Arginine	Arg	4.1	5.2	3.8	3.7	8.4	4.7	4.6	5.0	3.8	4.8	1.5
Isoleucine	Ile	3.9	5.2	3.2	4.5	6.3	4.3	4.3	5.0	3.2	4.4	1.0
Threonine	Thr	4.9	4.2	4.1	4.7	3.7	5.3	4.7	4.0	3.9	4.4	0.5
Phenylalanine	Phe	2.8	4.2	3.1	2.3	5.4	3.6	3.9	4.0	3.1	3.6	0.9
Asparagine	Asn	ND	3.1	ND	ND	ND	ND	ND	ND	ND	3.1	1.0
Tyrosine	Tyr	3.3	1.0	1.4	2.6	3.3	1.6	1.6	1.0	2.0	2.0	0.9
Lysine	Lys	1.1	ND	0.6	0.7	2.4	1.2	1.0	ND	0.6	1.1	0.7
Tryptophan	Trp	ND	ND	0.7	ND	ND	ND	ND	ND	ND	0.7	0.2
Methionine	Met	0.4	ND	0.1	1.0	1.7	0.3	0.5	ND	0.1	0.6	0.6
Histidine	His	0.5	ND	0.2	0.7	0.9	0.3	0.4	ND	0.2	0.5	0.3
Sum		100.0	100.0	99.9	100.0	99.4	100.0	100.0	96.0	90.9	107.0	

[(1)] When amino acid composition was given in mass units, the conversion to mole units was made assuming a molecular weight for keratin of 10 kDa. [(2)] The amino acid composition corresponds to three species of penguins. [(3)] The amino acid composition was given in the original work by the different parts of the feather. Here, the global composition is the simple mean value. [(4)] ND means not determined or not reported. Ref. 1[9]. Ref. 2[10]. Ref. 3[11]. Ref. 4[12]. Ref. 5[13]. Ref. 6[14]. Ref. 7[15]. Ref. 8[16]. They reported data from[17]. Ref. 9[18].

Table S3. Property values specified to create non-databank pseudo-components in Aspen Plus. The values shown were obtained by COSMO-RS calculations.

**Component**	**Molecular weight**	**Normal boiling temperature, °C**	**Density, kg/m³**
DES	202	406	1,301
SWK	1,454	1,845	1,012
ISK	1,440	1,486	964
KER	2,892	2,506	950

Table S4. Parameters A and B of the Andrade´s equation used in this work to specify $\eta = f(T)$ dependence on the individual components.

**Parameter**	**DES**	**SWK**	**ISK**	**KER**
A	-8.56	-12.1	-13.6	-20.6
B	4080.6	5694.4	6538.5	9083.5

Table S5. Information used to specify the COSMOSAC property model in Aspen Plus. Values were obtained by COSMOtherm v20.0 calculations on the molecular geometries optimized previously.

**Component**	**DES**	**SWK**	**ISK**	**KER**
**CSACVL, Å³**	226.5581	1769.1817	1719.7206	3438.9023
**Sigma-profiles**				
**Sigma. e/Å²**	**p(σ)**			
	**DES**	**SWK**	**ISK**	**KER**
-0.025	0	0	0	0
-0.024	0	0	0	0
-0.023	0	0	0	0
-0.022	0	0.018	0	0.018
-0.021	0	0.201	0	0.201
-0.020	0	0.528	0.129	0.657
-0.019	0.036	1.145	0.707	1.852
-0.018	0.762	2.273	1.907	4.180
-0.017	3.118	3.334	3.821	7.155
-0.016	6.409	3.854	5.964	9.818
-0.015	7.753	4.105	7.120	11.225
-0.014	6.502	4.364	7.030	11.394
-0.013	5.296	4.633	7.233	11.866
-0.012	5.128	5.343	8.285	13.628
-0.011	5.148	6.503	9.323	15.826
-0.010	5.028	9.273	14.151	23.424
-0.009	4.939	18.077	26.990	45.067
-0.008	4.893	31.847	40.848	72.695
-0.007	5.457	46.404	52.006	98.410
-0.006	6.614	61.047	70.246	131.293
-0.005	7.301	73.307	90.581	163.888
-0.004	7.157	81.779	101.207	182.986
-0.003	6.983	83.947	104.883	188.830
-0.002	7.663	82.786	103.623	186.409
-0.001	9.275	81.449	97.376	178.825
0	11.768	74.799	92.449	167.248

Table S5. Continuation.

Sigma. e/Å^2	p(σ) continuation			
	**DES**	**SWK**	**ISK**	**KER**
0.001	13.843	68.485	88.368	156.853
0.002	12.979	64.775	73.389	138.164
0.003	10.448	53.146	49.670	102.816
0.004	8.939	35.676	31.566	67.242
0.005	7.506	23.918	24.745	48.663
0.006	6.394	20.337	22.703	43.040
0.007	5.946	20.513	21.540	42.053
0.008	5.192	21.474	22.416	43.890
0.009	4.839	22.316	22.935	45.251
0.010	5.147	21.975	23.246	45.221
0.011	5.306	21.598	25.311	46.909
0.012	4.78	21.718	30.801	52.519
0.013	4.115	20.603	36.169	56.772
0.014	4.292	17.515	32.795	50.310
0.015	4.558	12.063	21.019	33.082
0.016	4.149	6.211	9.290	15.501
0.017	3.639	2.758	2.741	5.499
0.018	2.765	1.618	0.767	2.385
0.019	1.572	1.143	0.526	1.669
0.020	0.727	0.743	0.461	1.204
0.021	0.237	0.602	0.454	1.056
0.022	0.030	0.437	0.436	0.873
0.023	0	0.165	0.231	0.396
0.024	0	0.024	0.040	0.064
0.025	0	0	0	0

Table S6. *Dimensional* characteristics of the models created in this work to represent the keratin and the products of its decomposition.

Model structure	Global formula	No. atoms	Molecular weight	Degrees of freedom
Fragment (1)	$C_{62}H_{106}N_{18}O_{20}S$	207	1,454	615
Fragment (2)	$C_{61}H_{104}N_{18}O_{20}S$	204	1,440	606
Keratin	$C_{123}H_{208}N_{36}O_{40}S_2$	409	2,892	1,221

Table S7. Molecular and electronic properties of the molecular fragments (1) and (2) proposed in the current work to model the products of the keratin decomposition by interaction with the (NaAc + Urea) DES. COSMO-RS calculation.

Property	Fragment (1)	Fragment (2)
Molecular area, Å^2	1,140	1,387
Molecular volume, Å^3	1,769	1,720
H bond moment acceptor	17.5	25.2
H bond moment donor	8.7	10.6

Table S8. Heating necessities and utility costs related to the pre-heating of the reactor feed in the process proposed to dissolve and regenerate the keratin from chicken feathers (PRE-HEAT in Figure 2) with the (NaAc + urea) DES. The solvent added was considered the fresh (DES + water) mixture at 25 °C. The remainder specifications correspond to the Base Case (Table 1). Only the keratin dissolution section was considered.

**Reactor operating conditions**	**Heating fluid**	**Heating duty, kW**	**Operating cost, 10^3\$/o.y.**[(1)]
T = 80 °C, P = 1 bar	Medium pressure steam	2,673	170.9
T = 90 °C, P = 1 bar		3,198	204.1
T = 100 °C, P = 1 bar		3,733	238.0
T = 110 °C, P = 1 bar		4,279	272.6
T = 120 °C, P = 1 bar		4,834	307.8
T = 130 °C, P = 3 bar	High pressure steam	5,401	390.6
T = 140 °C, P = 3 bar		5,977	432.1
T = 150 °C, P = 3 bar		6,564	474.3
T = 160 °C, P = 3 bar		7,161	517.3
T = 170 °C, P = 4 bar		7,768	561.0

[(1)] *o.y.* means operational year.

Table S9. Equipment sizing of the keratin dissolution section in the process proposed to dissolve and regenerate the keratin from chicken feathers (Figure 2) with the (NaAc + Urea) DES. The solvent added was considered the fresh (DES + water) mixture at 25 °C. The remainder specifications correspond to the Base Case (Table 1). Only the keratin dissolution section was considered.

**Reactor operating conditions**	**Heating fluid**	**Reactor volume, m^3**	**PRE-HEAT area, m^2**	**Annuity, 10^3\$/o.y.**[(1)]
T = 80 °C, P = 1 bar	Medium pressure steam	5,056	67.0	926.8
T = 90 °C, P = 1 bar		1,913	96.2	486.8
T = 100 °C, P = 1 bar		757.1	124.2	264.2
T = 110 °C, P = 1 bar		314.6	159.3	149.2
T = 120 °C, P = 1 bar		137.6	194.9	88.1
T = 130 °C, P = 3 bar	High pressure steam	62.5	107.5	53.4
T = 140 °C, P = 3 bar		29.5	101.0	33.7
T = 150 °C, P = 3 bar		14.5	101.0	22.4
T = 160 °C, P = 3 bar		7.3	110.7	13.7
T = 170 °C, P = 4 bar		3.8	147.4	10.4

[(1)] *o.y.* means operational year.

Table S10. Volume reduction of the reactor used for dissolving the keratin from the chicken feathers with the (NaAc + Urea) DES. The feathers and the solvent were fed as fresh materials. $T_{REACTOR}$ = 120 °C. P = 1 bar. The remainder specifications correspond to the Base Case (Table 1). The volume reduction was evaluated respect to a single stirred tank operating at the same conditions (Table S9).

**Configuration of the reaction unit**	**Volume reduction, %**
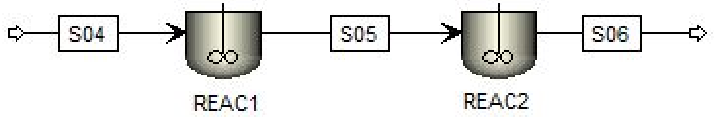	21.8
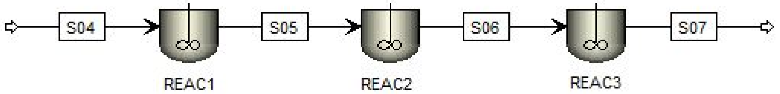	27.5
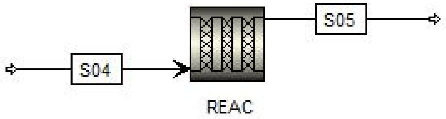	47.2

Table S11. Heat and material balances of the process proposed for the keratin dissolution and regeneration from chicken feathers using (NaAc + Urea) DES as reacting solvent. Process flowsheet is shown in Figure 2. Results correspond to the Base Case (Table 1) with no recycling of the regenerated solvent.

**Streams**	**S01**	**S02**	**S04**	**S06**	**S08**	**S09**	**S10**	**S11**	**S12**	**S13**	**S15**
VF[(1)], wt/wt	0	0	0	0	0	0	0	0.7	1	0	0
T, °C	25.0	25.0	25.5	120.0	60.0	60.0	60.0	127.1	127.1	127.1	127.1
P, bar	1	1	1	1	1	1	1	1	1	1	1
Mass flow, kg/h	2,500	125,000	127,500	127,500	293,250	415,947	4,804	415,946	290,984	124,962	124,962
**Composition, wt%**											
DES	0	90.0	88.2	88.2	0	27.0	0	27.0	0	90.0	90.0
SWK	0	0	0	0.6	0	0.2	0	0.2	0	0.6	0.6
IWK	0	0	0	0.6	0	0	15.5	0	0	0	0
KER	100	0	2.0	0.8	0	0	20.8	0	0	0	0
Water	0	10.0	9.8	9.8	100	72.8	63.6	72.8	100	9.4	9.4

(1) mass vapor fraction. T_{S05} = 120 °C. T_{S07} = 70 °C.

Table S12. Heat and material balances of the process proposed for the keratin dissolution and regeneration from chicken feathers using (NaAc + Urea) DES as reacting solvent. Process flowsheet is shown in Figure 2. Results correspond to the Alternative Case (Table 1) for which 60 wt% of the regenerated solvent (S13) is recycled (S14). The remainder specifications are those of the Base Case (Table 1).

**Streams**	**S01**	**S02**	**S04**	**S06**	**S08**	**S09**	**S10**	**S11**	**S12**	**S13**	**S15**
VF[(1)], wt/wt	0	0	0	0	0	0	0	0.7	1	0	0
T, °C	25.0	25.0	91.8	120.0	60.0	60.0	60.0	129.3	129.3	129.3	129.3
P, bar	1	1	1	1	1	1	1	1	1	1	1
Mass flow, kg/h	2,500	62,477	127,500	127,500	293,250	415,957	4,793	415,957	290,970	124,987	49,995
**Composition, wt%**											
DES	0	90.0	88.2	88.2	0	27.0	0	27.0	0	90.0	90.0
SWK	0	0	0.9	1.5	0	0.5	0	0.5	0	1.5	1.5
IWK	0	0	0	0.6	0	0	15.6	0	0	0	0
KER	100	0	2.0	0.8	0	0	20.9	0	0	0	0
Water	0	10.0	8.9	8.9	100	72.5	63.5	72.5	100	8.5	8.5

(1) mass vapor fraction. T_{S05} = 120 °C. T_{S07} = 70 °C.

Table S13. Heat and material balances of the process proposed for the keratin dissolution and regeneration from chicken feathers using (NaAc + Urea) DES as reacting solvent. Process flowsheet is shown in Figure 2. Results correspond to the Alternative Case (Table 1) for which 80 wt% of the regenerated solvent (S13) is recycled (S14). The remainder specifications are those of the Base Case (Table 1).

**Streams**	**S01**	**S02**	**S04**	**S06**	**S08**	**S09**	**S10**	**S11**	**S12**	**S13**	**S15**
VF[(1)], wt/wt	0	0	0	0	0	0	0	0.7	1	0	0
T, °C	25.0	25.0	116.5	120.0	60.0	60.0	60.0	134.4	134.4	134.4	134.4
P, bar	1	1	1	1	1	1	1	1	1	1	1
Mass flow, kg/h	2,500	25,053	127,500	127,500	293,250	415,978	4,737	415,978	291,044	124,934	24,987
**Composition, wt%**											
DES	0	90.0	88.2	88.2	0	27.0	0	27.0	0	90.0	90.0
SWK	0	0	2.4	3.0	0	0.9	0	0.9	0	6.0	6.0
IWK	0	0	0	0.6	0	0	15.6	0	0	0	0
KER	100	0	2.0	0.8	0	0	21.0	0	0	0	0
Water	0	10.0	7.4	7.4	100	72.1	63.4	72.1	100	4.0	4.0

(1) mass vapor fraction. T_{S05} = 120 °C. T_{S07} = 70 °C.

Table S14. Heat balance, as a function of the solvent recycled (SPLITT value), in the process proposed for the keratin dissolution and regeneration from chicken feathers using (NaAc + Urea) DES as reacting solvent. The utilities costs have also been added. Process flowsheets are shown in Figures 2 and S12. The remainder specifications correspond to the Base Case (Table 1). HEAT-WAT heating necessities are 11,913 kW.

Split	Heating/Cooling necessities, kW						$Q_{Heat.}/Q_{Cool.}$ (3)	Total util. cost, 10^3\$/o.y.(4)
	PRE-HEAT	COOLER	SEP-HEAT	COOL-S11(1)	COOL-S17(1)	Vapor in REGEN(2)		
0	4,835	-2,811	204,465	-89.7	-4,612	-187,562	4.5	5,507
0.1	4,317	-2,822	204,427	-89.7	-4,146	-187,512	4.7	5,466
0.2	3,796	-2,836	204,393	-89.7	-3,724	-187,665	4.9	5,416
0.3	3,271	-2,853	204,508	-89.7	-3,283	-187,734	5.1	5,379
0.4	2,741	-2,877	204,590	-89.6	-2,872	-187,834	5.4	5,339
0.5	2,204	-2,912	204,703	-89.6	-2,409	-187,813	5.7	5,307
0.6	1,638	-2,960	204,980	-89.6	-1,988	-187,926	6.1	5,278
0.7	1,018	-3,038	205,469	-89.6	-1,560	-188,020	6.5	5,261
0.8	235	-3,198	206,493	-89.6	-1,135	-188,514	6.8	5,249
Mean value	2,673	-2,923	204,892	-89.7	-2,859	-187,842	5.5	5,356

(1) Nomenclature corresponds to Figure S12. (2) The vapor produced at the REGEN is considered as a sub-product income. It is quoted as low pressure steam. (3) Ratio Heating necessities/Cooling necessities. (4) *o.y.* means operational year.

Table S15. Problem definition and exchanger geometry input specifications in the design of the heat exchangers with Aspen Exchanger Design and Rating (Aspen EDR). The overall heat transfer coefficients calculated by Aspen EDR for each heat exchanger is also given.

After the corresponding heat exchanger was calculated in Aspen Plus, the simulation data was exported to Aspen EDR. The following specifications and design criteria were used in Aspen EDR.

**Variable**	**Selected value/condition**
Calculation mode	Design
Location of hot fluid	Shell side
Allowable pressure drops	10-20% of the input pressure in each side. Maximum = 1 bar
Fouling resistance, m^2.K/W	Utility side = 0.0002<br>Process fluid side = 0.0003
TEMA type	BEM
Exchanger orientation	Horizontal
Tube outer diameter, mm	19.05
Tube thickness, mm	2.11
Tube layout	Pattern = 30-Triangular<br>Pitch = 23.81 mm
Baffles	Type = Single segmental<br>Orientation = Vertical
Design criteria	The lowest cost configuration satisfying the design specifications was taken as final design. No further revisions of the design were done due to its preliminary character

Overall heat transfer coefficients calculated for each heat exchanger by Aspen EDR. The current are the mean values for all the designs carried out.

**Heat exchanger**	**$U_{Mean\ Value}$(1), W/m^2.K**
PRE-HEAT	641.8
COOLER	531.3
SEP-HEAT	773.8
HEAT-WAT	1234.3
COOL-S11	190.0
COOL-S17	283.7
Inlet COOLER(2)	548.5
S06-S09 heat exchanger(3)	415.0

(1) *U* calculated include fouling. (2) When a S03:S01 mass ratio = 10 was used. (3) It was individually evaluated for the Base Case (Figure 2, Table 1) but was not finally incorporated to the process.

Table S16. Equipment sizing for different solvent recycle (SPLITT) values. The remainder specifications correspond to the Base Case (Table 1).

Split	Heat exchanger transfer area, m^2						Vessel volume, m^3		
	PRE-HEAT	COOLER	HEAT-WAT[(1)]	SEP-HEAT[(2)]	COOL-S11[(1)]	COOL-S17	REACTOR	MIX-DEC[(1)]	REGEN[(1,3)]
0	63.5	108.4	142.4	1,981	66.1	570.6	137.5	151.8	811.8
0.1	48.9	103.3		1,981		517.0	138.0		
0.2	44.0	107.3		1,981		491.0	138.6		
0.3	39.9	107.3		1,981		445.0	139.3		
0.4	28.3	107.3		1,981		364.1	140.3		
0.5	24.2	124.1		1,981		310.0	141.7		
0.6	20.1	108.4		1,981		260.5	143.6		
0.7	11.9	108.4		1,902		206.4	146.7		
0.8	5.5	135.5		2,105		103.0	152.7		

(1) No significant changes were observed with the solvent recycled. (2) 3 to 5 units (generally in parallel) were calculated as the best final design. (3) REGEN was oriented horizontally.

Table S17. Equipment costs for different solvent recycle (SPLITT) values. The remainder specifications correspond to the Base Case (Table 1).

Split	Equipment cost[(1)], 10^3\$								
	PRE-HEAT	COOLER	HEAT-WAT	SEP-HEAT	COOL-S11	COOL-S17	REACTOR	MIX-DEC	REGEN
0	28.0	31.1	39.0	692.1	23.4	109.4	754.9	230.9	105.9
0.1	22.5	33.1		692.1		95.3	755.6		
0.2	19.9	33.1		692.1		88.5	756.4		
0.3	19.2	33.1		692.1		80.3	757.6		
0.4	16.6	33.1		692.1		72.5	761.5		
0.5	16.0	33.5		692.1		63.3	766.4		
0.6	15.3	31.1		692.1		54.4	776.7		
0.7	14.2	31.1		716.0		46.5	783.4		
0.8	11.7	36.8		772.5		40.5	794.2		

(1) Materials of construction: SS-304 for vessels and CS for heat exchangers.

Table S18. Total costs summary for different solvent recycle (SPLITT) values for the process proposed to dissolve and regenerate the keratin from the chicken feathers using (NaAc + Urea) DES as reacting solvent (Figure 2). The remainder specifications in each case correspond to the Base Case (Table 1). These results are also shown in Figure 13SM.

Split	Annuities(1), 10^3\$/o.y.(2)	Utilities, 10^3\$/o.y.(2)	Total Cost, 10^3\$/o.y.(2)	Unitary cost(3), \$/t
0	201,5	5,507	5,708	285
0.1	199,8	5,467	5,666	283
0.2	198,9	5,416	5,614	281
0.3	198,1	5,380	5,578	279
0.4	197,5	5,339	5,537	277
0.5	197,0	5,307	5,504	275
0.6	196,9	5,278	5,475	274
0.7	199,0	5,261	5,460	273
0.8	205,5	5,250	5,455	273

(1) Annuities were calculated as the 10% of the equipment´s total purchasing costs. (2) *o.y.* means operational year. (3) Unitary costs were defined as the total (equipment´s purchased + utilities´) costs by ton of feathers treated.

Table S19. Heat and material balance of the process proposed for the keratin dissolution and regeneration from chicken feathers using (NaAc + Urea) DES as reacting solvent. Process flowsheet shown in Figure 2. Results correspond to the Alternative Case (Table 1) with S03:S01 = 10:1 and S08:S07 = 0.5:1. SPLITT = 0.6. The remainder specifications are those of the Base Case (Table 1).

**Streams**	**S01**	**S02**	**S04**	**S06**	**S08**	**S09**	**S10**	**S11**	**S12**	**S13**	**S15**
VF(1), wt/wt	0	0	0	0	0	0	0	0.4	1	0	0
T, ^oC	25	25	125.2	120.0	60.0	60.0	60.0	166.6	166.6	166.6	166.6
P, bar	1	1	1	1	1	1	1	1	1	1	1
Mass flow, kg/h	1,500	10,016	27,500	27,500	13,750	39,352	1,898	39,352	14,379	24,973	9,989
**Composition, wt%**											
DES	0	90.0	81.8	81.8	0	57.2	0	57.2	0.2	90.0	90.0
SWK	0	0	4.1	6.9	0	4.8	0	4.8	0	7.5	7.5
IWK	0	0	0	2.7	0	0	39.3	0	0	0	0
KER	100	0	9.1	3.6	0	0	52.7	0	0	0	0
Water	0	10.0	5.0	5.0	100	38.0	8.0	38.0	99.8	2.5	2.5

(1) mass vapor fraction. T_{S05} = 120 ^oC. T_{S07} = 70 ^oC.

Table S20. Heat and material balance of the process proposed for the keratin dissolution and regeneration from chicken feathers using (NaAc + Urea) DES as reacting solvent. Process flowsheet shown in Figure 2. Results correspond to the Alternative Case (Table 1) with S03:S01 = 20:1 and S08:S07 = 1.5:1. SPLITT = 0.6. The remainder specifications are those of the Base Case (Table 1).

**Streams**	**S01**	**S02**	**S04**	**S06**	**S08**	**S09**	**S10**	**S11**	**S12**	**S13**	**S15**
VF(1), wt/wt	0	0	0	0	0	0	0	0.6	1	0	0
T, ^oC	25.0	25.0	99.3	120.0	60.0	60.0	60.0	136.9	136.9	136.9	136.9
P, bar	1	1	1	1	1	1	1	1	1	1	1
Mass flow, kg/h	2,500	20,007	52,500	52,500	78,750	128,678	2,573	128,678	78,689	49,988	19,995
**Composition, wt%**											
DES	0	90.9	85.7	85.7	0	35.0	0	35.0	0	90.0	90.0
SWK	0	0	2.1	3.6	0	1.5	0	1.5	0	3.8	3.8
IWK	0	0	0	1.4	0	0	29.0	0	0	0	0
KER	100	0	4.8	1.9	0	0	38.9	0	0	0	0
Water	0	10.0	7.4	7.4	100	63.5	32.1	63.5	100	6.2	6.2

(1) mass vapor fraction. T_{S05} = 120 ^oC. T_{S07} = 70 ^oC.

Table S21. Heat and material balance of the process proposed for the keratin dissolution and regeneration from chicken feathers using (NaAc + Urea) DES as reacting solvent. Process flowsheet shown in Figure 2. Results correspond to the Alternative Case (Table 1) with S03:S01 = 30:1 and S08:S07 = 2.5:1. SPLITT = 0.6. The remainder specifications are those of the Base Case (Table 1).

**Streams**	**S01**	**S02**	**S04**	**S06**	**S08**	**S09**	**S10**	**S11**	**S12**	**S13**	**S15**
VF(1), wt/wt	0	0	0	0	0	0	0	0.7	1	0	0
T, ^{o}C	25.0	25.0	94.8	120.0	60.0	60.0	60.0	132.1	132.1	132.1	132.1
P, bar	1	1	1	1	1	1	1	1	1	1	1
Mass flow, kg/h	2,500	30,003	77,500	77,500	193,750	267,502	3,748	267,502	192,507	74,995	29,998
**Composition, wt%**											
DES	0	90.0	87.1	87.1	0	25.2	0	25.2	0	90.0	90.0
SWK	0	0	1.5	2.4	0	0.7	0	6.7	0	2.5	2.5
IWK	0	0	0	1.0	0	0	19.9	0	0	0	0
KER	100	0	3.2	1.3	0	0	26.7	0	0	0	0
Water	0	10.0	8.2	8.2	100	74.1	53.4	74.1	100	7.5	7.5

(1) mass vapor fraction. T_{S05} = 120 ^{o}C. T_{S07} = 70 ^{o}C.

Table S22. Heat balances for different S03:S01 and S08:S07 mass ratios. $T_{REACTOR}$ = 120 °C. The remainder specifications correspond to the Base Case (Table 1). 60% of the solvent regenerated is recycled (SPLIT = 0.6).

S03:S01 mass ratio	S08:S07 mass ratio	Heating and/or cooling necessities, kW							$Q_{Heat.}/Q_{Cool.}$(4)	Total util. cost, 10^3\$/o.y.(5)
		Heat cond.(1)	COOLER	SEP-HEAT	HEAT-WAT(2)	COOL-S11(2)	COOL-S17(2)	Vapor in REGEN(3)		
10	0.5	-81	-860	11,983	559	-22	-813	-9,543	1,7	383
	1.5	-81	-860	31,342	1,676	-28	-812	-27,709	3,0	844
	2.5	-81	-861	50,719	2,793	-35	-811	-49,300	2,4	1,118
20	0.5	542	-1,384	20,515	1,066	-25	-973	-17,325	2,0	642
	1.5	543	-1,384	56,624	3,197	-38	-973	-51,092	3,9	1,511
	2.5	543	-1,384	92,763	5,328	-50	-973	-84,862	5,7	2,382
30	0.5	921	-1,907	29,617	1,573	-29	-1,299	-25,263	2,1	923
	1.5	921	-1,907	82,744	4,719	-47	-1,298	-74,937	4,1	2,203
	2.5	921	-1,907	135,908	7,865	-65	-1,298	-124,607	6,1	3,485
40	0.5	1,280	-2,431	38,750	2,081	-32	-1,638	-33,203	2,2	1,206
	1.5	1,280	-2,432	108,905	6,242	-56	-1,638	-98,792	4,3	2,896
	2.5	1,280	-2,431	179,113	10,403	-80	-1,638	-164,380	6,4	4,590
50	0.5	1,633	-2,956	47,888	2,588	-36	-1,982	-41,196	2,2	1,485
	1.5	1,634	-2,956	135,076	7,764	-66	-1,982	-122,721	4,3	3,585
	2.5	1,634	2,956	222,328	12,940	-95	-1,982	-204,245	6.5	5,690

(1) It refers the heat conditioning of S04 to be feed to the reactor. For S03:S01 mass ratio 10:1 T_{SO4} > 120 ºC thus its cooling to the reactor operating temperature (120 °C) was necessary. On the contrary, when S03:S01 mass ratio was higher than 10, heating was required. (2) Nomenclature corresponds to Figure S12. (3) The vapor produced at the REGEN is considered as a sub-product income. It is quoted as low pressure steam. (4) Ratio Heating necessities/Cooling necessities. (5) *o.y.* means operational year.

Table S23. Equipment sizing for different S03:S01 and S08:S07 mass ratios. $T_{REACTOR}$ = 120 ^{o}C. The remainder specifications correspond to the Base Case (Table 1). 60% of the solvent regenerated is recycled (SPLIT = 0.6).

S03:S01 mass ratio	S08:S07 mass ratio	Heat exchanger transfer area, m^2						Vessel volume, m^3		
		Heat cond.(1)	COOLER	HEAT-WAT	SEP-HEAT	COOL-S11	COOL-S17	REACTOR	MIX-DEC	REGEN(2)
10	0.5	1.8	32.1	5.6	119.6	15.7	80.9	45.6	15.6	14.4
	1.5	1.8	32.1	16.7	312.8	20.5	80.8	45.6	25.1	57.2
	2.5	1.8	32.1	27.8	506.2	25.3	80.7	45.6	34.7	115.3
20	0.5	6.0	51.6	10.6	178.9	18.3	119.7	71.0	29.7	28.1
	1.5	6.0	51.6	31.9	493.9	27.5	119.7	71.0	47.9	130.3
	2.5	6.0	51.6	53.2	809.2	36.4	119.7	71.0	66.2	273.3
30	0.5	10.1	71.1	15.7	253.8	21.0	165.8	95.5	43.8	48.6
	1.5	10.1	71.1	47.1	709.1	34.3	165.8	95.5	70.8	225.2
	2.5	10.1	71.1	78.5	1,164.6	47.5	165.8	95.5	97.7	457.6
40	0.5	13.9	90.7	20.8	329.5	23.6	212.8	119.7	58.0	66.7
	1.5	13.9	90.7	62.3	926.1	41.1	212.8	119.7	93.6	327.8
	2.5	13.9	90.7	103.8	1,523.1	58.6	212.8	119.7	129.2	663.1
50	0.5	17.7	110.3	25.8	405.4	26.2	260.1	143.6	72.1	88.8
	1.5	17.7	110.3	77.5	1,143.5	47.9	260.1	143.6	116.4	457.6
	2.5	17.7	110.3	129.1	1,882.2	69.6	260.1	143.6	160.7	922.3

(1) For S03:S01 mass ratio 10:1 a cooler was calculated whereas a heater was used for the higher ratios. (2) REGEN was sized as a horizontal vessel.

Table S24. Equipment costs for different S03:S01 and S08:S07 mass ratios. $T_{REACTOR}$ = 120 °C. The remainder specifications correspond to the Base Case (Table 1). 60% of the solvent regenerated is recycled (SPLIT = 0.6).

S03:S01 mass ratio	S08:S07 mass ratio	Equipment cost[(1)], $10^3$$								
		Heat cond.[(1)]	COOLER	HEAT-WAT	SEP-HEAT	COOL-S11	COOL-S17	REACTOR	MIX-DEC[(2)]	REGEN[(3)]
10	0.5	8.2	15.0	5.6	128.4	9.9	26.9	338.2	50.7	19.8
	1.5	8.2	15.0	10.8	228.7	11.6	26.9	338.2	69.7	32.4
	2.5	8.2	15.0	14.7	305.2	13.1	26.9	338.2	86.4	42.8
20	0.5	7.5	20.0	8.2	163.6	10.8	34.1	454.2	77.9	24.9
	1.5	7.5	20.0	15.9	300.8	13.8	34.1	454.2	107.2	45.0
	2.5	7.5	20.0	21.6	404.5	16.3	34.1	454.2	132.8	61.9
30	0.5	10.2	24.2	10.4	201.7	11.7	41.5	553.3	101.0	30.5
	1.5	10.2	24.2	20.1	373.7	15.8	41.5	553.3	138.9	56.8
	2.5	10.2	24.2	27.3	503.2	19.2	41.5	553.3	172.1	78.2
40	0.5	12.3	28.0	12.3	235.9	12.6	48.1	642.8	121.6	34.4
	1.5	12.3	28.0	23.8	438.6	17.6	48.1	642.9	167.3	67.1
	2.5	12.3	28.0	32.3	591.2	21.7	48.1	642.7	207.3	93.1
50	0.5	14.2	31.5	14.0	267.2	13.4	54.3	725.7	140.7	38.5
	1.5	14.2	31.5	27.1	497.8	19.3	54.3	725.7	193.4	78.2
	2.5	14.2	31.5	36.8	671.2	24.1	54.3	725.7	239.7	109.2

[(1)] Materials of construction: SS-304 for vessels and CS for heat exchangers.

Table S25. Total costs for the Base and different Alternative Cases (Table 1) of the process proposed to dissolve and regenerate the keratin from the chicken feathers using (NaAc + Urea) DES as reacting solvent. 60% of the DES regenerated is recycled. The remainder specifications in each case correspond to the Base Case (Table 1).

S03:S01 mass ratio	S08:S07 mass ratio	Annuities[(1)], 10^3\$/o.y.[(2)]	Utilities, 10^3\$/o.y.[(2)]	Total cost, 10^3\$/o.y.[(2)]	Unit. cost[(3)], \$/t
10	0.5	60	383	443	22,1
	1.5	74	844	918	45,9
	2.5	85	1118	1204	60,2
20	0.5	80	642	722	36,1
	1.5	100	1511	1610	80,5
	2.5	115	2382	2497	124,8
30	0.5	98	923	1022	51,1
	1.5	123	2203	2326	116,3
	2.5	143	3485	3628	181,4
40	0.5	115	1206	1321	66,0
	1.5	145	2896	3040	152,0
	2.5	168	4590	4757	237,9
50	0.5	130	1485	1615	80,8
	1.5	164	3585	3749	187,5
	2.3	186	5269	5455	277,8
	2.5	191	5690	5880	294.0

(1) Annuities are calculated as the 10% of the equipment´s total purchasing cost. (2) *o.y.* means operational year. (3) Unitary costs are defined as the total (equipment´s purchased costs + utilities´) costs by ton of feathers treated.

Table S26. Dependence of the total equipment´s and utilities´ costs for the Base and different Alternative Cases (Table 1) of the process proposed to dissolve and regenerate the keratin from the chicken feathers using (NaAc + Urea) DES as reacting solvent with both the solvent (S03:S01 mass ratio) and water (S08:S07 mass ratio) excesses used. 60% of the regenerated DES is recycled. The remainder specifications in each case correspond to the Base Case (Table 1). The total costs are decomposed by process sections (Figure 2).

S03:S01 mass ratio	S08:S07 mass ratio	Equipment costs				Utility costs			
		Total cost, 10^3\$	Relative contributions, %			Total cost, 10^3\$/o.y.	Relative contributions, %		
			Ker. dissol.	Ker. regen.	DES recov.		Ker. dissol.	Ker. regen.	DES recov.
10	0.5	603	57,5	13,5	29,1	383	0,1	9,5	90,4
	1.5	741	46,7	14,4	38,8	844	0,1	11,6	88,4
	2.5	850	40,7	15,2	44,1	1118	0,0	14,2	85,8
20	0.5	801	57,6	14,6	27,8	642	6,1	10,5	83,4
	1.5	998	46,2	15,7	38,1	1511	2,6	12,2	85,2
	2.5	1153	40,0	16,5	43,4	2382	1,6	12,6	85,7
30	0.5	984	57,2	15,0	27,8	923	7,2	10,7	82,1
	1.5	1234	45,7	16,1	38,2	2203	3,0	12,3	84,7
	2.5	1429	39,4	17,0	43,6	3485	1,9	12,7	85,4
40	0.5	1148	57,1	15,2	27,7	1206	7,6	10,8	81,6
	1.5	1446	45,3	16,4	38,3	2896	3,2	12,4	84,5
	2.5	1677	39,1	17,3	43,7	4590	2,0	12,8	85,2
50	0.5	1299	56,9	15,4	27,7	1485	7,9	10,8	81,2
	1.5	1641	45,1	16,5	38,4	3585	3,3	12,4	84,3
	2.3	1860	39,8	17,2	43,0	5269	2,2	12,7	85,0
	2.5	1907	38,8	17,4	43,8	5690	2,1	12,8	85,1

Table S27. Heat and material balance of the process proposed for the keratin dissolution and regeneration from chicken feathers using (NaAc + Urea) DES as reacting solvent. Process flowsheet shown in Figure 2. Results correspond to the Alternative Case (Table 1) with S03:S01 = 20:1, S08:S07 = 1:1 and SPLITT = 0.6 which was taken as the final design in the current work. The remainder specifications are those of the Base Case (Table 1).

**Streams**	**S01**	**S02**	**S04**	**S06**	**S08**	**S09**	**S10**	**S11**	**S12**	**S13**	**S15**
VF(1), wt/wt	0	0	0	0	0	0	0	0.5	1	0	0
T, °C	25.0	25.0	99.3	171.4	60.0	60	60	136.9	136.9	136.9	136.9
P, bar	1	1	1	4	1	1	1	1	1	1	1
Mass flow, kg/h	2,500	20,005	52,500	52,500	52,500	102,690	2,310	102,690	52,698	49,992	19,997
**Composition, wt%**											
DES	0	90.0	85.7	85.7	0	43.8	0	43.8	0	90.0	90.0
SWK	0	0	2.1	3.6	0	1.9	0	1.9	0	3.8	3.8
IWK	0	0	0	1.4	0	0	32.3	0	0	0	0
KER	100	0	4.8	1.9	0	0	43.3	0	0	0	0
Water	0	10.0	7.4	7.4	100	54.3	24.4	54.3	100	6.2	6.2

(1) mass vapor fraction. T_{S05} = 130 °C. T_{S07} = 70 °C.

Table S28. Heat balances (in kW) of the process proposed for the keratin dissolution and regeneration from chicken feathers using (NaAc + Urea) DES as reacting solvent. Process flowsheet shown in Figure 2. Results correspond to the Alternative Case (Table 1) with S03:S01 = 20:1, S08:S07 = 1:1 and SPLITT = 0.6, which was taken as the final design in the current work. The remainder specifications are those of the Base Case (Table 1).

**Heat cond.**(1)	**PUMP**(2)	**COOLER**	**SEP-HEAT**	**HEAT-WAT**(3)	**COOL-S11**(3)	**COOL-S17**(3)	**Vapor in REGEN**(4)	**Total util. cost, 10^3\$/o.y.**(5)
816.3	8.9	-4,700	38,590	2,133	-31.4	-974	-34,239	1,121

(1) It refers the heating of S04 to be feed to the reactor, $T_{In,REACTOR}$ = 130 °C. (2) S05 was pumped to the operating pressure of the reactor, $P_{Reactor}$ = 4 bar. In this table the electricity power is given. Pump and driver efficiencies of 0.75 and 0.95 were set in the calculations. (3) For nomenclature, see Figure 12SM. (4) The vapor produced by head of the REGEN was considered as a sub-product income. It was quoted as low pressure steam. (5) *o.y.* means operational year.

Table S29. Equipment sizing of the process proposed for the keratin dissolution and regeneration from chicken feathers using (NaAc + Urea) DES as reacting solvent. Process flowsheet shown in Figure 2. Results correspond to the Alternative Case (Table 1) with S03:S01 = 20:1, S08:S07 = 1:1 and SPLITT = 0.6, which was taken as the final design in the current work. The remainder specifications are those of the Base Case (Table 1).

Heat exchanger transfer area, m^2						Vessel volume, m^3			Power, kW
**PRE-HEAT**	**COOLER**	**HEAT-WAT**[(1)]	**SEP-HEAT**	**COOL-S11**[(1)]	**COOL-S13**[(1)]	**REACTOR**[(2)]	**MIX-DEC**	**REGEN**[(3)]	**PUMP**[(4)]
14.4	143.5	31.6	334.6	26.2	119.9	5.5	38.8	66.7	8.9

[(1)] For nomenclature, see Figure S12. [(2)] It was sized as a tube reactor (PFR model in Aspen Plus) oriented horizontally. [(3)] REGEN was sized as horizontal vessel. [(4)] S05 was pumped to the operating pressure of the reactor, $P_{Reactor}$ = 4 bar. Pump and driven efficiencies were set to 0.75 and 0.95, respectively. Power is given as final electricity consumed.

Table S30. Equipment costs of the process proposed for the keratin dissolution and regeneration from chicken feathers using (NaAc + Urea) DES as reacting solvent. Process flowsheet shown in Figure 2. Results correspond to the Alternative Case (Table 1) with S03:S01 = 20:1, S08:S07 = 1:1 and SPLITT = 0.6, which was taken as the final design in the current work. The remainder specifications are those of the Base Case (Table 1).

Equipment cost[(1)], 10^3\$									
**PRE-HEAT**	**COOLER**	**HEAT-WAT**[(2)]	**SEP-HEAT**	**COOL-S11**[(2)]	**COOL-S13**[(2)]	**REACTOR**[(3)]	**MIX-DEC**	**REGEN**[(4)]	**PUMP**[(5)]
13.3	38.6	16.2	131.9	15.8	34.9	96.2	93.1	34.4	8.6

[(1)] Materials of construction: SS-304 for vessels and CS for heat exchangers. [(2)] For nomenclature, see Figure S12. [(2)] It was quoted as jacketed horizontal vessel. [(4)] REGEN was quoted as horizontal vessel. [(5)] It was mapped as a centrifugal pump.

Table S31. Cost summary of the Alternative Case (Table 1) with S03:S01 = 20:1, S08:S07 = 1:1 and SPLITT = 0.6 (which was taken as the final design in the current work) for the process proposed to the keratin dissolution and regeneration from chicken feathers using (NaAc + Urea) DES as reacting solvent. Process flowsheet shown in Figure 2. The remainder specifications correspond to the Base Case (Table 1).

Purchased equipment cost[(1)], 10^3\$	Utility cost, 10^3\$/o.y.	Total cost, 103\$/o.y.[(2)]	Unitary cost[(3)], \$/t
483	1,121	1,170	58.5

[(1)] Annuities, in 10^3\$/o.y. are computed as the 10% of the total equipment cost. [(2)] *o.y.* means operational year. [(3)] Unitary cost is given by ton of chicken feathers treated.

References

1. Nuutinen, E.-M.; Willberg-Keyriläinen, P.; Virtanen, T.; Mija, A.; Kuutti, L.; Lantto, R.; Jääskeläinen, A.-S., Green process to regenerate keratin from feathers with an aqueous deep eutectic solvent. *RSC Adv.* **2019,** 9, (34), 19720-19728.

2. Zhang, Z.; Nie, Y.; Zhang, Q.; Liu, X.; Tu, W.; Zhang, X.; Zhang, S., Quantitative change in disulfide bonds and microstructure variation of regenerated wool keratin from various ionic liquids. *ACS Sustain. Chem. Eng.* **2017,** 5, (3), 2614-2622.

3. Idris, A.; Vijayaraghavan, R.; Patti, A. F.; MacFarlane, D. R., Distillable protic ionic liquids for keratin dissolution and recovery. *ACS Sustain. Chem. Eng.* **2014,** 2, (7), 1888-1894.

4. Ji, Y.; Chen, J.; Lv, J.; Li, Z.; Xing, L.; Ding, S., Extraction of keratin with ionic liquids from poultry feather. *Sep. Purif. Technol.* **2014,** 132, 577-583.

5. Ghosh, A.; Clerens, S.; Deb-Choudhury, S.; Dyer, J. M., Thermal effects of ionic liquid dissolution on the structures and properties of regenerated wool keratin. *Polym. Degrad. Stab.* **2014,** 108, 108-115.

6. Idris, A.; Vijayaraghavan, R.; Rana, U. A.; Fredericks, D.; Patti, A. F.; MacFarlane, D. R., Dissolution of feather keratin in ionic liquids. *Green Chem.* **2013,** 15, (2), 525-534.

7. Li, R.; Wang, D., Preparation of regenerated wool keratin films from wool keratin-ionic liquid solutions. *J. Appl. Polym. Sci.* **2013,** 127, (4), 2648-2653.

8. Wang, Y.-X.; Cao, X.-J., Extracting keratin from chicken feathers by using a hydrophobic ionic liquid. *Process Biochem.* **2012,** 47, (5), 896-899.

9. Zhao, W.; Yang, R.; Zhang, Y.; Wu, L., Sustainable and practical utilization of feather keratin by an innovative physicochemical pretreatment: high density steam flash-explosion. *Green Chem.* **2012,** 14, (12), 3352.

10. Arai, K. M.; Takahashi, R.; Yokote, Y.; Akahane, K., Amino-acid sequence of feather keratin from fowl. *Eur. J. Biochem.* **1983,** 132, (3), 501-7.

11. Gregg, K.; Rogers, G. D., Chapter 33: Feather keratin. Composition, structure and biogenesis. *In: Bereiter-Hahn J., Matoltsy A.G., Richards K.S. (eds) Biology of the Integument. Springer, Berlin, Heidelberg* **1986**.

12. Murphy, M. E.; King, J. R.; Tarusco, T. G.; Guepel, G. R., Amino acid composition of feather barbs and rachises in three species of pygoscelid penguins. Nutritional implications. *Condor* **1990,** 92, 913-921.

13. Sangali, S.; Brandelli, A., Feather keratin hydrolysis by a vibrio *sp.* strain kr2. *J. Appl. Microbiol.* **2000,** 89, 735-743.

14. Akahane, K.; Murozono, S.; Murayama, K., Soluble proteins from fowl feathers keratin. *J. Biochem.* **1977,** 81, 11-18.

15. Nuutinen, E.-M., Feather characterization and processing. *Master´s Thesis. Aalto University. School of Chemical Engineering* **2017**.

16. Martinez-Hernandez, A. L.; Velasco-Santos, C.; de Icaza, M.; Castano, V. M., Microstructural characterisation of keratin fibres from chicken feathers. *Int. J. Environ. Pollut.* **2005,** 23, (2), 162-178.

17. Schmidt, W. F., Innovative feather utilization strategies. *Proceeding Poultry Waste Management Conference. Springdale, Arkansas* **1998**.

18. Harrap, B. S.; Woods, E. F., Soluble derivatives of feather keratin. 1. Isolation, fraction and amino acid composition. *Biochem. J.* **1964,** 92, (1), 8-18.
